# Applying systems thinking to unravel the mechanisms underlying orthostatic hypotension related fall risk

**DOI:** 10.1007/s11357-023-00802-9

**Published:** 2023-04-28

**Authors:** Liping Wang, Anouschka C. Pronk, Eveline P. van Poelgeest, Robert Briggs, Jurgen A.H.R. Claassen, Sofie Jansen, Marjolein Klop, Frederik J. de Lange, Carel C.G.M. Meskers, Vincent J. J. Odekerken, Stephen J. Payne, Marijke C. Trappenburg, Roland D. Thijs, Jeroen F. Uleman, Alfons G. Hoekstra, Nathalie van der Velde

**Affiliations:** 1 Amsterdam UMC location University of Amsterdam, Internal Medicine, Geriatrics, Meibergdreef 9, Amsterdam, The Netherlands; 2 Amsterdam Public Health, Aging and Later Life, Amsterdam, The Netherlands; 3 The Irish Longitudinal Study on Ageing, Trinity College Dublin, Dublin, Ireland; 4 Department of Biophysics, Donders Institute for Brain, Cognition and Behaviour, Radboud University, Nijmegen, The Netherlands; 5 Amsterdam UMC location University of Amsterdam, Cardiology and Cardiothoracic Surgery, Meibergdreef 9, Amsterdam, The Netherlands; 6 Amsterdam UMC location Vrije Universiteit Amsterdam, Rehabilitation Medicine, De Boelelaan, 1117 Amsterdam, The Netherlands; 7 Amsterdam Movement Sciences, Amsterdam, The Netherlands; 8 Amsterdam UMC location University of Amsterdam, Neurology, Meibergdreef 9, Amsterdam, The Netherlands; 9 Institute of Applied Mechanics, National Taiwan University, Taipei, Taiwan; 10 Department of internal medicine, Ziekenhuis Amstelland, Amstelveen, The Netherlands; 11 Department of Neurology, Leiden University Medical Centre, Leiden, The Netherlands; 12 Stichting Epilepsie Instellingen Nederland (SEIN), Heemstede, The Netherlands; 13 Department of Geriatric Medicine, Donders Institute for Brain, Cognition and Behaviour, Radboud University Medical Center, Nijmegen, The Netherlands; 14 Institute for Advanced Study, Amsterdam, The Netherlands; 15 Computational Science Lab, Informatics Institute, Faculty of Science, University of Amsterdam, Amsterdam, The Netherlands

**Keywords:** Orthostatic hypotension, Falls, Older adults, Geriatrics, Causal loop diagram, Group model building

## Abstract

Orthostatic hypotension (OH) is an established and common cardiovascular risk factor for falls. An in-depth understanding of the various interacting pathophysiological pathways contributing to OH-related falls is essential to guide improvements in diagnostic and treatment opportunities. We applied systems thinking to multidisciplinary map out causal mechanisms and risk factors. For this, we used group model building (GMB) to develop a causal loop diagram (CLD). The GMB was based on the input of experts from multiple domains related to OH and falls and all proposed mechanisms were supported by scientific literature. Our CLD is a conceptual representation of factors involved in OH-related falls, and their interrelatedness. Network analysis and feedback loops were applied to analyze and interpret the CLD, and quantitatively summarize the function and relative importance of the variables. Our CLD contains 50 variables distributed over three intrinsic domains (cerebral, cardiovascular, and musculoskeletal), and an extrinsic domain (e.g., medications). Between the variables, 181 connections and 65 feedback loops were identified. Decreased cerebral blood flow, low blood pressure, impaired baroreflex activity, and physical inactivity were identified as key factors involved in OH-related falls, based on their high centralities. Our CLD reflects the multifactorial pathophysiology of OH-related falls. It enables us to identify key elements, suggesting their potential for new diagnostic and treatment approaches in fall prevention. The interactive online CLD renders it suitable for both research and educational purposes and this CLD is the first step in the development of a computational model for simulating the effects of risk factors on falls.

**Supplementary Information:**

The online version contains supplementary material available at 10.1007/s11357-023-00802-9.

## Introduction

Falls among older adults are a major and increasing public health problem. Annually, one-third of individuals over the age of 65 falls at least once, and 20% of these falls lead to severe injuries [1–3]. Falls are often multifactorial. In unexplained and recurrent falls, cardiovascular diseases are relatively frequently present, but not always recognized [4, 5].

Orthostatic hypotension (OH) is an established and one of the most common cardiovascular risk factors for falls [6, 7]. Thus, as recommended by the recently published World Guidelines on Falls Prevention, assessment and treatment of OH is a standard component of the multifactorial fall prevention approach [4]. A recent systematic review and meta-analysis [8] showed an almost doubled risk of falls in older adults for (OR 1.73; 95% CI 1.50–1.99). The incidence of OH increases with age and has been shown to contribute to up to one-third of the fall incidents in older individuals [6, 7, 9]. Analogous to falls, OH has a multifactorial etiology [10, 11], making OH-related falls a particularly complex health concern. The best-known pathway from OH to falls involves the direct effect of inadequate brain perfusion upon standing [12]. However, various other contributing mechanisms are involved and needs to be considered in older adults. For example, cerebral white matter lesions (resulting from recurrent episodes of cerebral hypoperfusion due to OH) may lead to motor dysfunction or cognitive impairment, which in turn contribute to falls indirectly [13]. Also, OH is linked to poorer physical functioning, which is a fall risk factor [14]. However, most studies in the field are observational in nature, and generally focused on single pathophysiological routes. A comprehensive understanding of the interactions between causes is lacking and these studies do not capture the complex interactions of other intrinsic and extrinsic risk factors superimposed on the normal aging process of the individual. An in-depth understanding of the various interacting pathophysiological pathways contributing to OH-related falls is essential to identify critical fall preventive factors and such an overarching understanding requires interdisciplinary collaboration between disciplines that now mainly focus on single pathophysiological routes.

Applying systems thinking offers a methodology to understand the behavior of complex systems [15–18] and thus be helpful in unraveling the interactions and pathways between OH and falls. An important concept in systems thinking is the causal loop diagram (CLD) [18]: a conceptual model of relevant mechanisms and interactions developed by experts in the field, which can highlight the dynamic nature of an issue and help explore the multiple, interacting feedback mechanisms within a system of interest [19]. This can lead to an improved understanding of a complex system, such as OH-related falls. Thus, the aim of this study was to develop an expert consensus CLD on OH-related falls, by combining and weighing existing and evolving evidence of the processes involved and to identify those factors with the highest fall preventive potential for use in clinical practice.

## Methods

### Group model building

Our CLD was developed by following the structure of Group Model Building (GMB) approach, a participatory system thinking approach in which experts engage in the process of developing conceptual or computational models [20–22]. In GMB, the perceptions and knowledge of experts are elicited and captured in a shared model, which is the result of consensus in the group, and the resulting model is a summary of explicit, tested, and integrated knowledge of the group [17, 21, 22]. For the formation of our (national and international) expert group (Supplementary Table 1), we identified the clinical areas of knowledge relevant to OH-related falls, namely internal medicine, geriatrics, physiology, clinical pharmacology, rehabilitation medicine, cardiology, and neurology as well as methodological experts from the field of computational science, knowledgeable on the clinical subject. We aimed to include a variety of expertise, but also ensure some overlap. The experts were derived from established groups, namely the Dutch Syncope Society and the European Geriatric Medicine Society (EuGMS) Special Interest Group on Falls and Fractures. Methodological experts (computational science) were invited through the network of consortium members. This resulted in a total of 16 national and international experts. The study was conducted according to the principles in the Declaration of Helsinki. The study was exempted from ethical approval as there was no infringement of the physical and/or psychological integrity of the participants. Before participating in the project, the experts received written information about the background, aims, and process of the project. Consent to participate was given before entering the project.

First, we held three general GMB sessions resulting in the backbone structure of the CLD. Based on the first findings, the variables were categorized into three intrinsic domains (cerebral, cardiovascular, and musculoskeletal), and one extrinsic domain that included variables devoid of incoming connections (including relevant medications, temperature, and alcohol variables). Subsequently, nine domain-specific subgroup sessions were organized based on the main research topics of the experts. The meetings were organized over the course of one year (2021), with several weeks in between. Length of the meetings varied between 1.5 and 3 h.

**Fig. 1:**
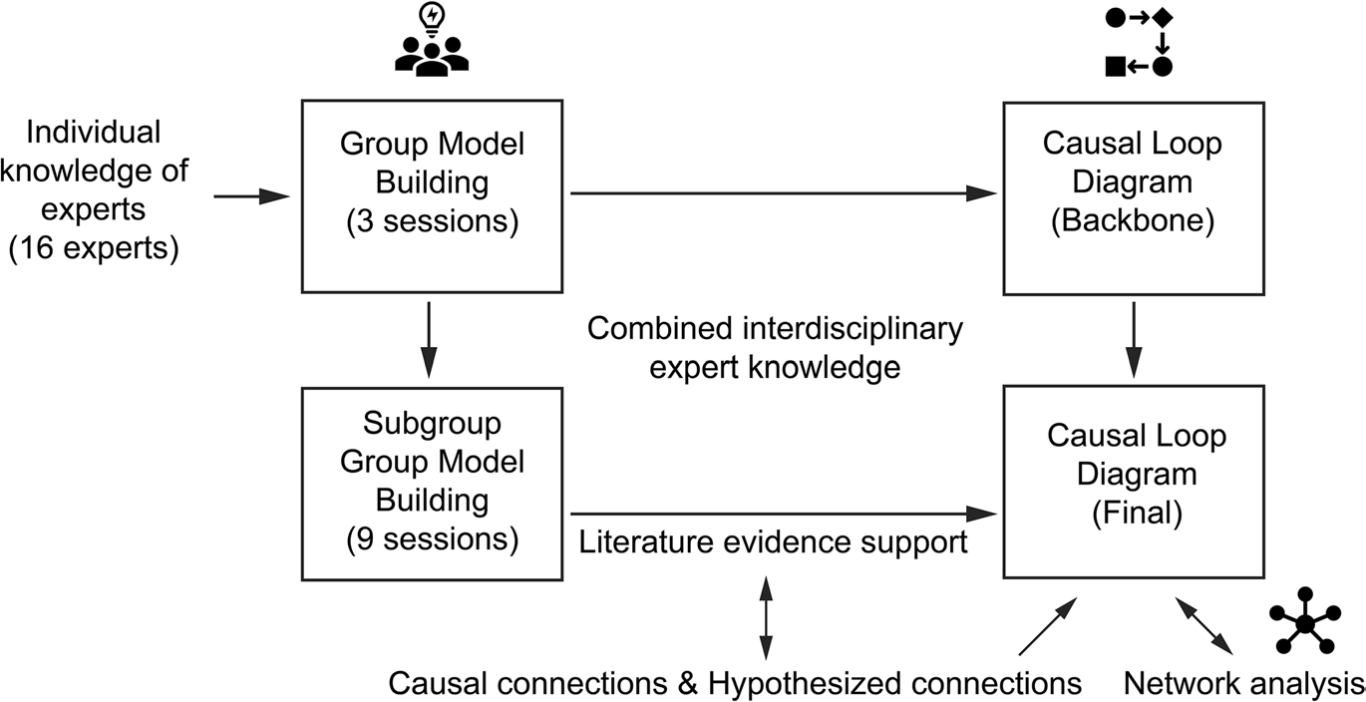
Schematic representation of the process for group model building to develop a causal loop diagram

During the GMB meetings, a facilitator led the GMB discussions, and a computational modeler sketched the connections in the CLD (Vensim, Ventana Systems Inc. 2022) in real time. A graphical representation of the GMB approach for developing a CLD is shown in Fig. 1. First, each expert was encouraged to propose three variables the expert deemed most relevant, and to suggest how these variables would be linked to other variables in the CLD. After that, a plenary discussion followed until a consensus was reached on the new variables and connections. All connections were confirmed by scientific evidence from the literature, where after each meeting experts were asked to provide relevant literature for their included variables and connections and a comprehensive literature search was conducted to find relevant literature for the included variables and connections. If after reviewing the published literature and consultations with the experts there remained uncertainties about the validity of connections, the connections were considered hypothetical.

### Causal loop diagram

A CLD is a graphical representation of different pathways describing the factors (called nodes) and their interrelatedness (called connections, both known and hypothesized) relevant to complex problems under study [18, 23]. Positively connected variables in the CLD (displayed as “+”) follow the same direction: when a causal variable increases, the variable it is linked to also increases; if the variable decreases, its linked variable also decreases. Negatively connected variables (“−”) have an opposite direction; when a causal variable increases, the variable it is linked to decreases, and vice versa [18, 24]. For some connections, the polarity can change depending on certain conditions. For example, the negative polarity from blood pressure to stroke volume can change when blood pressure gets too high (Frank-Starling mechanism) [25]. In general, when heart rate increases, stroke volume increases as well. However, when heart rate increases too high, stroke volume decreases [26]. Connections like these have double polarity in the CLD (“+/−”).

Feedback loops are important features within a CLD [18]. Reinforcing feedback loops (displayed as “R”) accelerate/strengthen change and potentially disturb the system, whereas balancing feedback loops (“B” sign) counteract change and promote stabilization of the system [18, 23, 24]. Hypothesized loops (“H” sign) can be either reinforcing or balancing but contain connections for which evidence from the literature is limited or evolving. Loops up to length 5 were identified, using Vensim [24], and the potential clinical relevance was described in a narrative in the results section. Also, an interactive (visualization) of the CLD was created in Kumu (2022; https://kumuio/2022) [27] to allow visual inspection and analysis of the CLD, and to easily navigate the CLD and the underlying scientific evidence for variables and links between variables.

### Network analysis

The resulting CLD is represented as a graph or network of relationships among a set of variables and thus can be interpreted to form a network structure. Network analysis provides a suite of quantitative techniques that can summarize the structure of a network and quantify the importance of its elements [27, 28]. Network analysis on the CLD may facilitate the identification of the key drivers in CLDs by quantifying their structural importance in the system [27]. Although the identification of feedback loops can be seen as a form of analyzing the network [28], betweenness centrality (BC) and closeness centrality (CC) are frequently used measures for network analysis [29]. BC measures the extent to which each variable (node) lies on the shortest paths between other variables (nodes) in the network [27, 30]. High BC variables might therefore have a mediating function, making them potential targets for interventions [18, 29–31]. As a complementary, CC measures how close a variable (node) is to the other variables (nodes) in a network [30]. It may relate to the speed or efficiency with which one variable connects to other variables [29, 31]. Variables with high CC have the shortest distance to many other variables, suggesting that they may rapidly exert their effects in the network. Variables with both high BC and CC may play a central role in the CLD and could be relevant to inform potential intervention approaches [18, 29]. Analysis and interactive visualization of the network analysis was conducted using the Kumu [], which applies well-established algorithms for computing network statistics, based on the algorithm for calculating shortest paths provided by Freeman and Brandes [30–32].

## Results

**Fig. 2:**
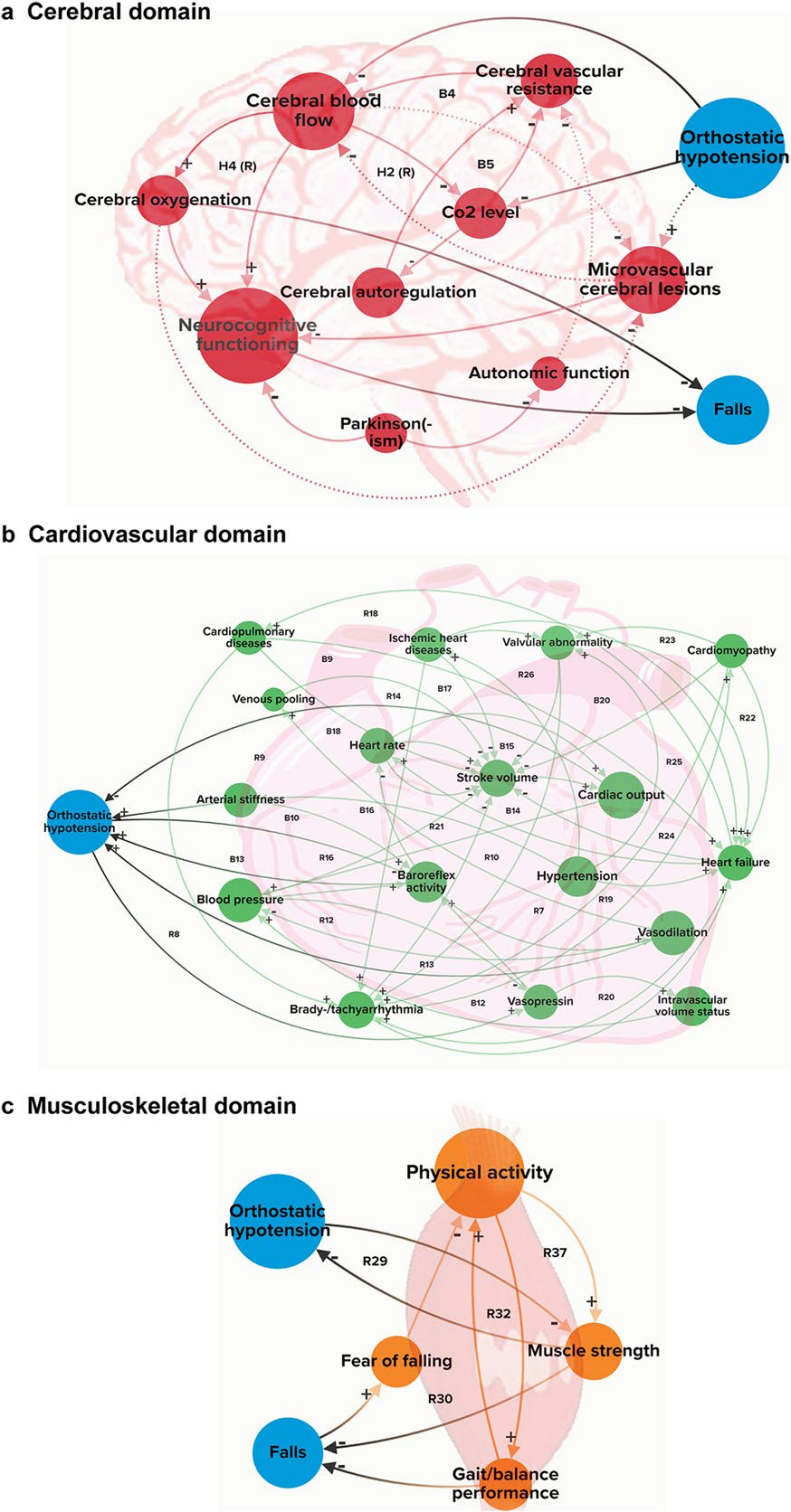
Three main intrinsic domains (cerebral, cardiovascular, and musculoskeletal) of causal loop diagram for orthostatic hypotension-related falls. The variables of the diagram were categorized into three intrinsic domains: cerebral (in red), cardiovascular (in green), and musculoskeletal (in orange) based on the organ system, and the two key variables (in blue), with the (causal) connections between these variables. A positive connection (+) represents an effect in the same direction, e.g., an increase/decrease in “*X*” causes similar change in “*Y*,” whereas a negative connection (−) represents an effect in the opposite direction, e.g., an increase/decrease in “*X*” causes opposite change in “*Y*.” A hypothesized connection is shown as a dotted line. Reinforcing feedback loops are indicated with “R,” balancing feedback loops with “B” and feedback loops that contained hypothesized (dotted) connection with “H.” The size of the variables is scaled by their betweenness centrality. An online interactive version is available at https://fallscld.kumu.io/understanding-the-multicausality-between-orthostatic-hypotension-and-falls-19a96c2e-a7a1-47b0-a3bf-53d6ea837dde

**Fig. 3:**
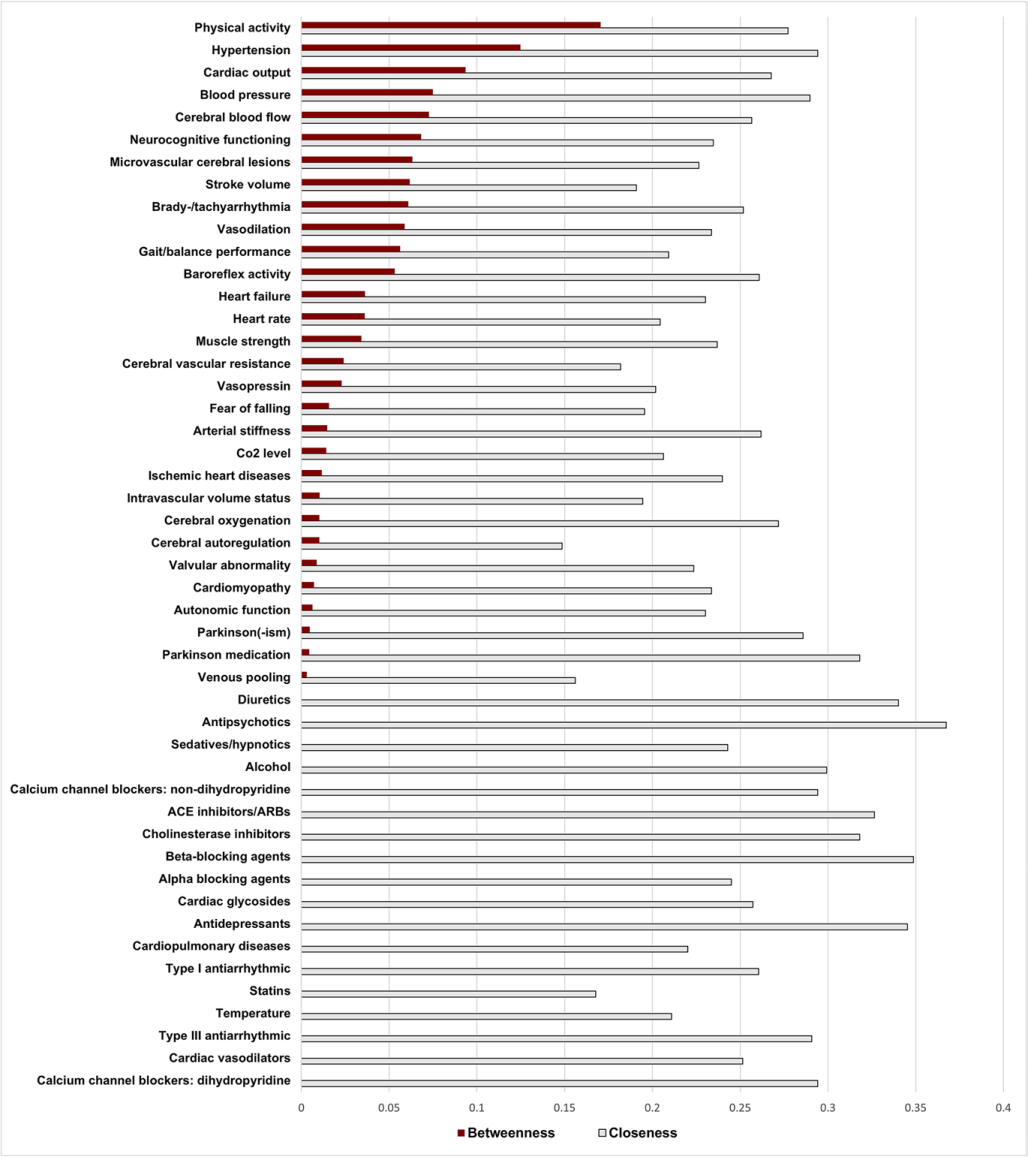
The betweenness and closeness centrality for the variables in the causal loop diagram. The variables are ranked descending based on their betweenness centrality results

Our CLD contains 50 variables and 181 connections between them (Supplementary Fig. 1). An interactive version of the CLD can be found online (https://fallscld.kumu.io/understanding-the-multicausality-between-orthostatic-hypotension-and-falls-19a96c2e-a7a1-47b0-a3bf-53d6ea837dde). Three main intrinsic domains (cerebral, cardiovascular, and musculoskeletal) of causal loop diagram for orthostatic hypotension-related falls are shown in Fig. 2. Variable definitions and supporting literature evidence for the connections can be found in Supplementary Tables 2 and 3. We identified 65 feedback loops (37 reinforcing, 21 balancing, and 7 hypothesized) in the CLD. An overview of all the identified feedback loops can be found in Supplementary Table 4. An overview of betweenness and closeness centrality for the variables in the CLD is shown in Fig. 3. In this section, we first describe the main physiological balance system and then report the results from three intrinsic domains (cerebral, cardiovascular, and musculoskeletal), and one extrinsic domain.

**Fig. 4:**
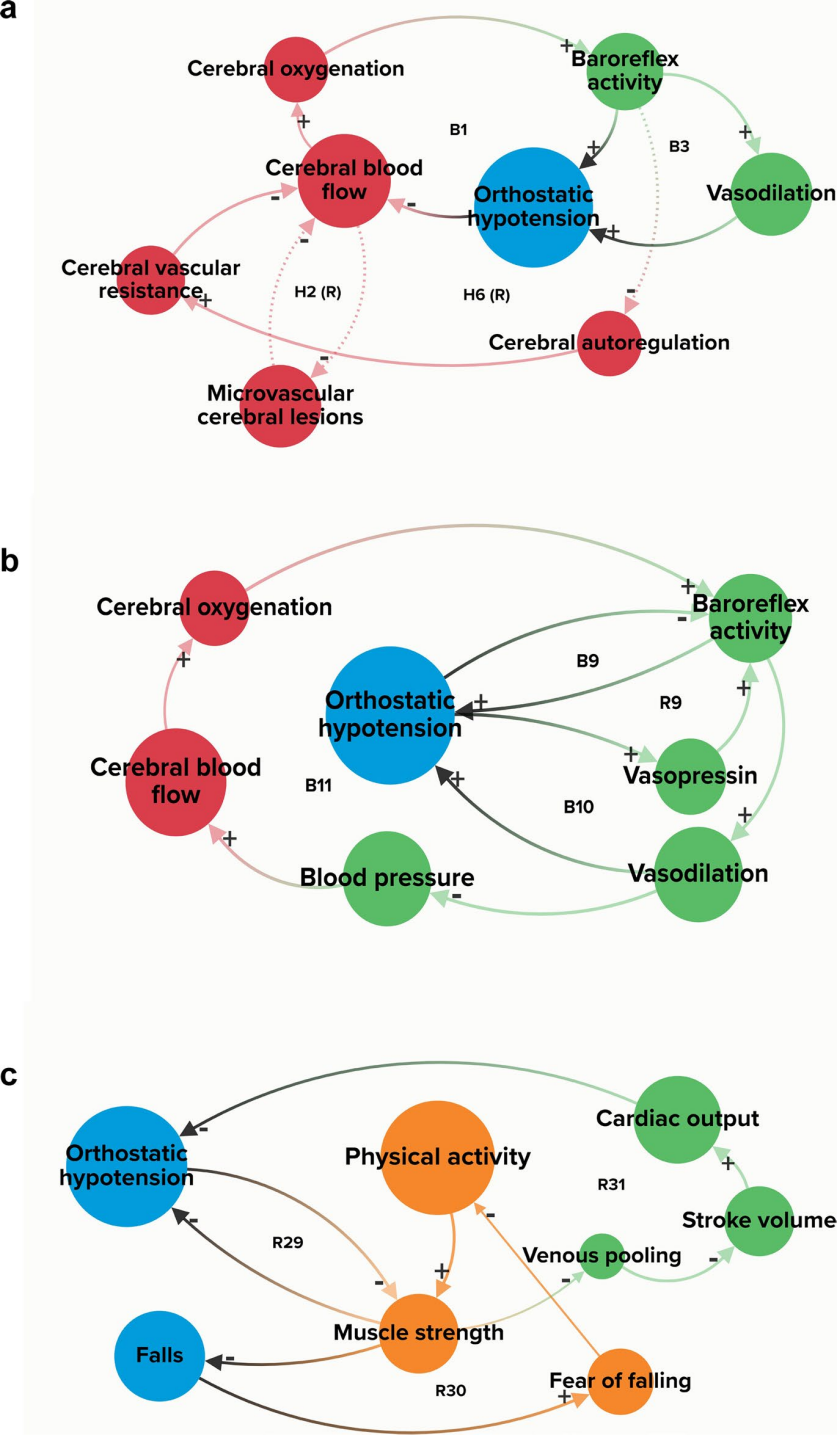
**a**–**c** Examples of some key feedback loops in orthostatic hypotension-related falls

The main physiological balancing systems that ensure the maintenance of cerebral perfusion upon standing are shown in B1, B3, and B11 (Fig. 4a, b and Supplementary Table 4). A change from supine to standing position leads to pooling of blood (up to 1L) in the lower extremities and splanchnic vasculature, decreasing venous return and stroke volume and subsequently causing a drop in blood pressure. In order to restore blood pressure and maintain adequate cerebral blood flow, different balancing physiological/homeostatic effects act in concert [33]: when blood pressure drops due to orthostatic challenge/gravitational effects, baroreceptors, as part of the short-term blood pressure regulation, are unloaded to restore blood pressure (B9, Fig. 4b and Supplementary Table 4). Also, with (orthostatic) blood pressure drops, cerebral blood flow decreases, and cerebral oxygenation decreases, triggering diminished baroreflex firing, resulting in less vasodilation, therefore reducing the occurrence of OH (B9, B10, B11, Fig. 4b and Supplementary Table 4).

### Cerebral domain

In the cerebral domain, cerebral blood flow has high BC (Fig. 3), connecting to various variables within and across domains (Fig. 4 and Supplementary Table 3 and 4). Given its high centrality, decreased cerebral blood flow could be a central factor from the cerebral domain in OH-related falls and may play a vital role in (pre)syncope. Cerebral blood flow directly influences cerebral oxygenation (positive polarity) with a (negative) connection to falls. When compensatory mechanisms are adequate, however, a decrease in cerebral oxygenation does not lead to a fall (e.g., loop B1, B10-B11, Supplementary Table 4).

Similarly, cerebral autoregulation is the mechanism that aims to stabilize or restore cerebral blood flow when there are changes in blood pressure. As can be seen in the CLD, baroreflex activity, blood pressure, arterial stiffness, and carbon dioxide (CO_2_) influence cerebral autoregulation. A decrease in cerebral blood flow decreases cerebral oxygenation, which decreases baroreflex activity, and consequently (hypothetically) activates cerebral autoregulation (hypothesized reinforcing loop H6, Fig. 4a, Supplementary Table 4). In turn, this increases cerebral vascular resistance, which further decreases cerebral blood flow (static autoregulation). It is controversial whether older age by itself is related to decreased function of cerebral autoregulation. Although mixed data have been published, there is some evidence suggesting that with aging, cerebral autoregulation is negatively affected, but that these changes (if any) are only small [34, 35]. With older age, baroreflexes become less sensitive and there is slowing of blood pressure recovery [34, 36], rendering older adults to be more vulnerable for blood pressure drops than their younger counterparts, especially when they are dehydrated and/or use vasodilating medications (e.g., nitrates and alpha blocking agents). As a result, it puts older adults at greater risk of changes in cerebral blood flow and consequently falls. In addition, carbon dioxide (CO_2_) is well-known for exerting potent cerebral blood flow responses [40] (B4 and B5, Supplementary Table 4), but its role is highly complex, and related to the speed in which blood pressure drops evolve.

Several hypothesized connections (involved in feedback loops) in the cerebral domain were proposed by experts. For instance, microvascular cerebral lesions may also contribute to OH-related falls (hypothetical H1–H5, Supplementary Table 4). In H2 (Fig. 4a), there is a direct reinforcing feedback loop from cerebral blood flow to microvascular lesions, which presents the short-term/immediate negative effect of reduced cerebral blood flow (due to a drop in blood pressure) [37, 38]. Hypothetical loop H4 presents the long-term effect where repeatedly decreased cerebral oxygenation causes microvascular cerebral lesions, which negatively influences neurocognitive functioning [39, 40]. A recent prospective study, however, did not confirm that OH resulted in white matter lesions [41]. Cerebral lesions and cognitive disorders are also associated with fall risk [42].

### Cardiovascular domain

In the cardiovascular domain, hypertension is a variable with high BC and CC (Fig. 3). As such, it may play an essential role in OH-related falls. Noticeably, baroreflex activity is involved in multiple feedback loops (Supplementary Table 4), both reinforcing and balancing of the short-term blood pressure regulation. The central role of baroreflex activity and its engagement in multiple feedback loops makes it vulnerable to disruption of the system. For instance, in R9 (Fig. 4b; Supplementary Table 4), the orthostatic blood pressure drop stimulates release of vasopressin, which in turn increases baroreflex activity and results in blood pressure restoration [43–45]. In B11 (Fig. 4b; Supplementary Table 4), a blood pressure drop decreases cerebral blood flow and cerebral oxygenation. Baroreflex activity decreases and vasoconstriction in turn restores blood pressure. Inadequate baroreflex effects can have a significant contribution to OH-related falls. Multiple variables in our CLD (e.g., arterial stiffness, autonomic nervous system failure, and medications) can negatively influence baroreflex functioning. For example, in autonomic failure, compensatory increase in heart rate and vasoconstriction are diminished/inadequate, potentially leading to OH. As seen in the CLD, cardiovascular diseases/conditions can have disruptive (reinforcing) effects when concomitantly present (R18–R26, Supplementary Table 4).

### Musculoskeletal domain

In the musculoskeletal domain, physical activity shows high centrality with the highest BC and high CC (Fig. 3). As a “mediator that connects the different organ systems,” physical inactivity influences both OH and falls through muscle strength, gait/balance performance, and cardiovascular variables (e.g., heart rate and cardiac output). Also note that physical activity is part of all the feedback loops in the musculoskeletal domain. All identified feedback loops in this domain (Supplementary Table 4) are reinforcing and highly interrelated, implicating that changes in variables have the potential to disrupt or strengthen the system, not only in the musculoskeletal domain, but also in other domains. Physical activity and muscle strength play a central role in the musculoskeletal domain of our CLD and have close connections to factors from the other domains. For example, in R31 (Fig. 4c; Supplementary Table 4), decreased muscle strength (e.g., in sarcopenia) negatively affects OH by increased/prolonged venous pooling, resulting in decreased stroke volume and cardiac output; in turn, this reinforces the development of OH. Similarly, R29 is nested in R31, indicating muscle strength also has a reinforcing negative influence on OH (Fig. 4c; Supplementary Table 4). Furthermore, R30 (Supplementary Table 4) shows how falls can negatively influence fall risk through changes in muscle strength: falls can lead to fear of falling [46, 47], and consequently, more physical inactivity with further loss of muscle mass [48].

### Extrinsic domain

The extrinsic domain contains different medications, temperature, and alcohol intake. Most of the extrinsic variables have relatively high CC compared to the intrinsic variables, suggesting that the system can be influenced by external factors. Specifically, medications (e.g., antipsychotics, antidepressants, and beta-blocking agents) have high CC (Fig. 3), indicating that they may be contributors to OH-related falls. Moreover, medications, alcohol, and temperature influence various variables in our CLD across all three intrinsic domains. For instance, high temperature causes vasodilation and vasopressin release, both involved in cerebral blood flow feedback loops (B3, B8, and R6, Supplementary Table 4). In addition, medications with vasodilating properties and alcohol can promote venous pooling or vasodilation contributing to OH (R31, Fig. 4c). Considering these variables are “extrinsic” to the system, they may be modifiable risk factors and thus can be viewed as promising starting points for interventions (e.g., performing a medication review, and switching culprit medications to safer alternatives).

## Discussion

We developed a comprehensive conceptual model of 50 variables involved in OH-related falls and identified which of these are most relevant. In the cerebral domain, we identified cerebral blood flow as a key factor in OH-related falls based on its high centrality. Cerebral hypoperfusion and reduced cerebral oxygenation contribute to OH and (pre)syncopal symptoms including falls [49]. There are several noninvasive diagnostic techniques to measure these variables. For example, cerebral blood flow can be assessed with transcranial Doppler, and cerebral oxygenation can be measured through near-infrared spectroscopy (NIRS). With NIRS, information related to regional cerebral blood flow and oxygenation can be captured real time [50]. For example, in patients with unexplained syncope, NIRS measurements showed a significant decrease in frontal cerebral tissue oxygenation saturation during the head-up tilt test, and loss of consciousness when cerebral tissue oxygenation fell below 60% [51]. Whether NIRS measurements are of benefit in falls prevention needs to be confirmed.

In the cardiovascular domain, we identified blood pressure as an essential “mediator” in OH-related falls based on its high BC. On the one hand, hypotension can cause or aggravate OH, but the same is true for uncontrolled hypertension [52]. Uncontrolled hypertension also increases the risk of cardiovascular complications (e.g., myocardial infarction and heart failure with reduced ejection fraction) [53]. Therefore, hypertension should be adequately treated in older patients, even in those at risk of falling. This was illustrated by the SPRINT trial [54], in which intensive blood pressure lowering was shown to be effective in preventing major cardiovascular events also demonstrated in the older (>75 years) participants with hypertension. In their study group, targeting systolic blood pressure to <120 mmHg appeared safe, without increasing the risk of (injurious) falls or syncope [55]. This was also demonstrated in the STEP study [56]. However, these studies excluded the frailer older adults [56]. In TILDA (The Irish Longitudinal Study on Aging) cohort (≥75 years of age), the authors demonstrated a 5-fold higher rate of injurious falls/syncope in participants who did not meet the SPRINT inclusion criteria compared to the relatively healthy SPRINT participants [56]. Data from observational studies consistently show that too strict hypertension treatment may lead to OH and OH-related falls [6].

In concurrence with this, a recent systematic review demonstrated that withdrawal of antihypertensive medications in older people is safe [57]. In line with this, a non-randomized trial demonstrated that in frail older adults, OH can be improved by deprescribing antihypertensives, resulting in an reduction in OH-related falls risk [58]. It is therefore important to make personalized decisions in patients with hypertension and take patient characteristics such as frailty into account [59]

Besides blood pressure, we also indicated the importance of impaired baroreflex activity as a key element in OH-related falls literature [43–45, 60]. Baroreflex activity is linked to stroke volume and heart rate. Because common age-related diseases/conditions (e.g., heart failure, autonomic failure [44]) and commonly used medications in older adults (e.g., beta-blockers) have effects on stroke volume and/or heart rate, baroreflex activity may be an important target for fall preventive interventions in older adults.

We identified that in the musculoskeletal domain, all feedback loops involved physical activity, and that muscle strength and gait/balance performance are important contributing factors in OH-related falls proposed by experts. This is not surprising, as physical counter maneuvers (e.g., leg-crossing and squatting) are well-established cornerstones of OH management, and exercise interventions (especially those targeting gait, balance, and muscle strength) have been proven their efficacy in falls prevention [61, 62]. Literature suggests that increased muscle tensing likely reduces OH [61–63], but further research is necessary to confirm this.

### Strengths and limitations

To our knowledge, we are the first to develop a comprehensive CLD within fall research. This approach is relatively novel in medical science and particularly suitable for answering complex clinical research questions [18, 21] as needed to study falls prevention in older adults. We characterized the complex pathophysiological pathways (and their interrelatedness) involved in OH-related falls. We combined evidence from the literature with expert knowledge from the fields of internal medicine, geriatrics, physiology, clinical pharmacology, cardiology, neurology, rehabilitation medicine, and computational science as input for our CLD. We applied network analysis to summarize and quantify the structure of our CLD, and thus generated original data. In the field of falls prevention, we are the first to develop a CLD and perform network analysis to quantify the strength of the connections and rank the importance of the variables. Our findings add to the knowledge base that until now has been confirmed to single pathways [8, 14]. To the best of our knowledge, in geriatric medicine, CLDs have only been developed in the field of cognition [18, 64]. In their CLD paper on Alzheimers’s disease, Uleman et al. also conducted network analysis and feedback loop analysis to rank the importance of variables and further analyze their CLD [18].

Our study has several limitations. First, our aim was to capture and understand the most important mechanisms between OH and falls, based on the expertise in our project group. As a result, this CLD may not be a complete conceptual model of the topic. Second, the most important variables in this conceptual model are mainly determined based on static structural features (network analysis), which may not relate to the variables’ causal and dynamic importance in physiology. In addition, the CLD methodology, without translating into a stock and flow model, is unable to account for time-dependent variations [15], whereas these variations are relevant to the pathways in our model. For example, cerebral autoregulation depends on the speed of blood pressure changes (slow blood pressure changes are more effectively buffered than fast changes, whereas extremely fast changes cannot be buffered at all) [32].

### Implications for clinical practice and future directives

Our CLD provides a comprehensive overview of the complex and multifactorial pathways involved in OH-related falls. The CLD is freely accessible online (https://fallscld.kumu.io/understanding-the-multicausality-between-orthostatic-hypotension-and-falls-19a96c2e-a7a1-47b0-a3bf-53d6ea837dde) and interactive in nature: by hovering, pathways of interest can be selected, while providing literature evidence for them. Therefore, our CLD can be used by healthcare professionals to reason about patients’ etiology and can also be used to explain to patients how their individual risk factors contribute to their OH-related falls risk, and how this risk can be mitigated by interventions aimed at reducing OH [19, 21].

Our CLD can also inform researchers in the field of falls prevention on the most promising targets for interventions aimed at reducing fall risk in older people. This CLD can be considered a contemporary starting point for the research community. The field is evolving rapidly, however, and evidence on OH-related falls continues to increase [8, 65]. We encourage colleagues to enhance and update our CLD based on the latest evidence, for example on our hypothesized connections/pathways, especially in the cerebral domain (e.g., the role of measuring cerebral blood flow in falls prevention) [34, 51].

We used this CLD to identify the centrality of variables involved in OH-related falls and analyzed these variables mainly qualitatively with feedback loops [16, 18, 27, 66]. Next, we will develop a quantitative systems dynamics model of this CLD [17]. This model will enable us to capture the dynamics of the processes related to OH and falls and estimate the effect size of fall preventive intervention strategies [67–69].

## Conclusion

This CLD shows the relevance and feasibility of applying systems thinking and GMB to capture the complex and multifactorial pathophysiology of OH-related falls by combining state-of-the-art knowledge from the fields of geriatric medicine, neurology, cardiology, physiology, rehabilitation medicine, and clinical pharmacology. Our CLD increased our understanding of this highly complex and major health care problem. We identified cerebral blood flow, blood pressure, baroreflex activity, and physical activity as the key elements in OH-related falls, suggesting their potential for new diagnostic and treatment approaches in fall prevention. Our CLD combines and adds to existing knowledge on single pathways and feedback mechanisms that do not fully account for the interacting behavior of a multicausal network. This CLD will be used for the development of a system dynamics model, which means it can become an important tool for simulating and predicting falls.

### Supplementary information

ESM 1

## Data Availability

The authors declare that the data supporting the findings of this project are available within the article and its supplementary files.
