# Supplementary material for: Applying systems thinking to unravel the mechanisms underlying orthostatic hypotension related fall risk
Source: GeroScience. 2023 Apr 28;45(4):2743–55. doi: 10.1007/s11357-023-00802-9 (PMC10651607; doi:10.1007/s11357-023-00802-9)
Supplement: Supplementary file 1 — ESM 1 [file 11357_2023_802_MOESM1_ESM.docx]

**Applying systems thinking to unravel the mechanisms underlying orthostatic hypotension related fall risk**

## **Supplementary Table 1 Members of the research project (in alphabetical order).**

| **Member** | **Fields of expertise** |
| --- | --- |
| Robert Briggs, MD, PhD | Geriatric medicine, falls and syncope |
| Jurgen Claassen, MD, PhD | Geriatric medicine, falls and syncope, physiology, dementia, hypertension |
| Alfons Hoekstra, PhD, Prof. | Computational science |
| Sofie Jansen, MD, PhD | Geriatrics, cardiovascular falls and syncope |
| Marjolein Klop, PhD student | Geriatric medicine, orthostatic hypotension, falls and syncope, physiology |
| Freek de Lange, MD, PhD | Cardiology, syncope |
| Carel Meskers, MD, PhD, Prof. | Rehabilitation medicine, orthostatic hypotension |
| Vincent Odekerken, MD, PhD | Neurology, movement disorders |
| Stephen Payne, PhD, Prof. | Cerebral blood flow, physiology, biomedical modelling |
| Eveline van Poelgeest, MD, PhD | Internal medicine, geriatrics, falls prevention, clinical pharmacology |
| Anouschka Pronk, MD, PhD student | Internal medicine, geriatrics, falls prevention |
| Marijke Trappenburg, MD, PhD | Internal medicine, geriatric medicine, falls and syncope |
| Roland Thijs, MD, PhD | Neurology, autonomic failure |
| Jeroen Uleman, PhD student | Computational science, system dynamic modelling |
| Nathalie van de Velde, MD, PhD, Prof. | Internal medicine, geriatrics, falls prevention |
| Liping Wang, PhD student | Cardiovascular falls prevention |

**Supplementary Fig. 1 Causal loop diagram of orthostatic hypotension-related falls.**


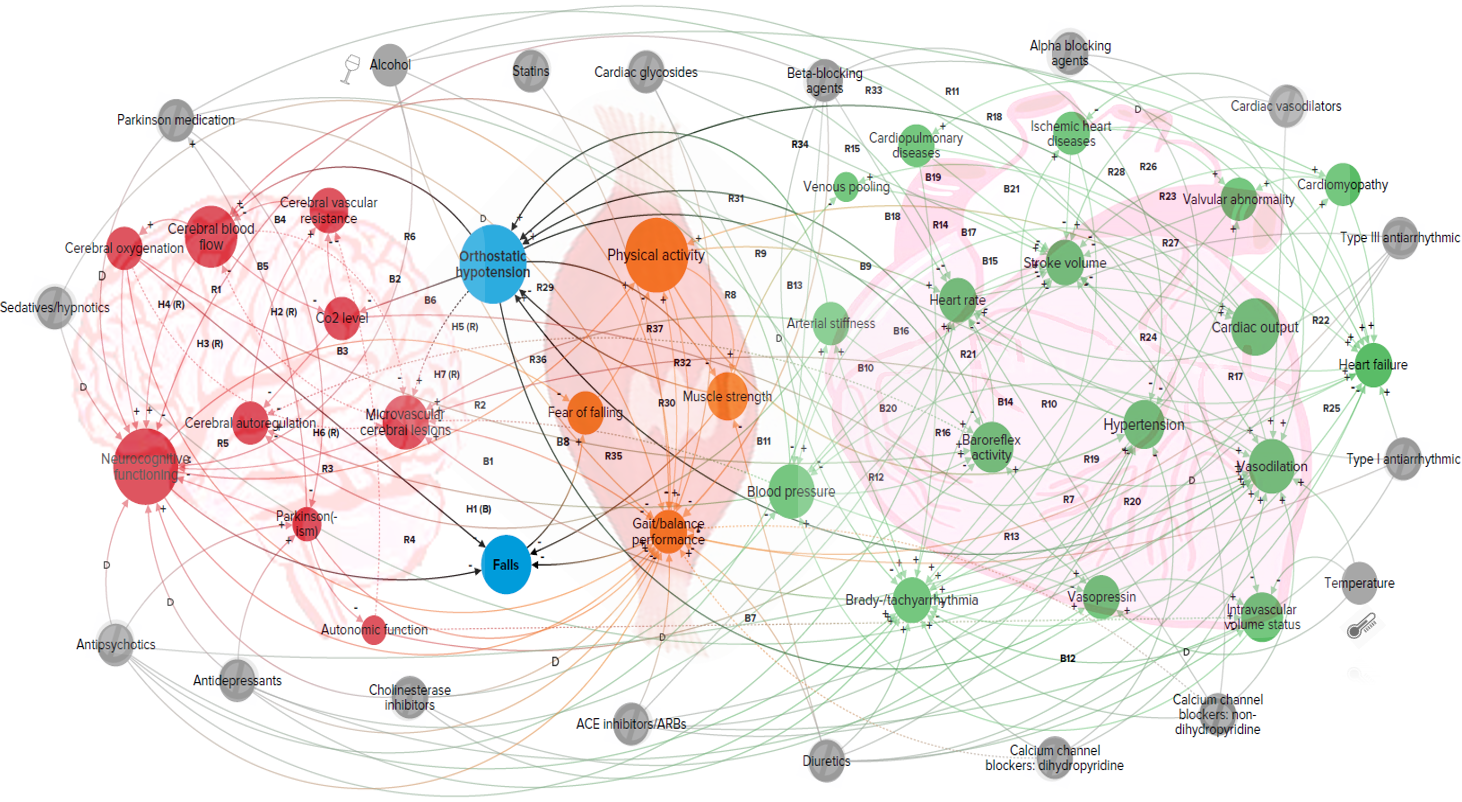


The diagram consists of 50 variables, categorized into three intrinsic domains: cerebral (in red), cardiovascular (in green), and musculoskeletal (in orange), one extrinsic domain (in grey), and the two key variables (in blue), and 181 (causal) connections between the variables. A positive connection (+) represents an effect in the same direction, e.g., an increase/decrease in ‘X’ causes similar change in ‘Y’; whereas a negative connection (-) represents an effect in the opposite direction, e.g., an increase/decrease in ‘X’ causes opposite change in ‘Y’. A hypothesized connection is shown as a dotted line. Reinforcing feedback loops are indicated with ‘R’, balancing feedback loops with ‘B’ and feedback loops that contained hypothesized (dotted) connection with ‘H’. The size of the variables is scaled by their betweenness centrality. An online interactive version is available at: <https://fallscld.kumu.io/understanding-the-multicausality-between-orthostatic-hypotension-and-falls-19a96c2e-a7a1-47b0-a3bf-53d6ea837dde>

**Supplementary Table 2** Definitions of included variables.

| **Domain** | **Variable** | **Definition** |
| --- | --- | --- |
| **Key variables** | Falls | An unexpected event in which the individual comes to rest on the ground, floor, or lower level |
|  | Orthostatic hypotension (OH) | A drop in blood pressure of at least 20mmHg in systolic blood pressure and/or 10 mmHg in diastolic blood pressure within 5 minutes after standing up, also known as postural hypotension. Orthostatic hypotension can also occur within 30 seconds of standing (initial orthostatic hypotension), or after 3 minutes of standing (delayed OH) |
| **Cerebral domain** | Cerebral blood flow | A measurement of brain perfusion (volume of blood that flows per unit mass per unit time through the brain tissue) |
|  | Cerebral oxygenation | Cerebral oxygen (O_2_) delivery and utilization |
|  | Cerebral autoregulation | The ability of the cerebral vasculature to maintain adequate cerebral blood flow despite changes in blood pressure (cerebral perfusion pressure) |
|  | Cerebrovascular resistance | The ratio of cerebral perfusion pressure (e.g. the difference between mean arterial pressure and intracranial pressure) to cerebral blood flow; the cerebral vascular network regulates blood flow distribution by adjusting vessel diameters, and consequently resistance to flow in response to changes in perfusion pressure and metabolic demands |
|  | CO2 level | Partial pressure of carbon dioxide in blood (PCO_2_) |
|  | Neurocognitive functioning | Mental abilities in perceptual-motor function, language, learning and memory, social cognition, complex attention and executive function |
|  | Autonomic function | Peripheral nervous system functioning in regulation of involuntary physiologic processes (e.g. heart rate, blood pressure, respiration, digestion) |
|  | Microvascular cerebral lesions | Lesions in the brain resulting from small blood vessel conditions, including white matter lesions, lacunar infarcts and microbleeds |
|  | Parkinson(-ism) | Parkinson's disease, Parkinson-like disorders (e.g. dementia with Lewy bodies, progressive supranuclear palsy, multiple system atrophy), and drug-induced parkinsonism |
| **Cardiovascular domain** | Baroreflex activity | Activity of the baroreflex responses-homeostatic mechanisms working in concert to maintain short term blood pressure regulation, heart rate and blood volume within a narrow physiologic range in reaction to short term circulatory changes and body posture. |
|  | Cardiac output (CO) | The amount of blood the heart pumps through the circulatory system in one minute (formula: cardiac output = stroke volume x heart rate) |
|  | Stroke Volume | The volume of blood pumped from the left ventricle per single beat |
|  | Venous Pooling | Pooling of blood in the veins of the lower extremities and splanchnic regions |
|  | Blood pressure | Arterial blood pressure. Includes both systolic and diastolic blood pressure |
|  | Heart rate | The number of heart beats per minute |
|  | Intravascular volume status | The volume of blood in the blood vessel compartment |
|  | Vasopressin | Arginine vasopressin (AVP), a neuropeptide secreted by the pituitary gland, also known as antidiuretic hormone |
|  | Vasodilation | Increase in arterial or venous vessel diameter caused by relaxation of smooth muscle cells in the vessel walls |
|  | Arterial stiffness | Impaired arterial distensibility associated with endothelial dysfunction, for example in hypertension |
|  | Hypertension | Long-term elevated blood pressure |
|  | Brady/tachyarrhythmia | Disturbance of heart rhythm with abnormally fast OR abnormally slow ventricular rate |
|  | Ischemic heart diseases | Heart disease resulting from coronary artery atherosclerosis resulting in myocardial ischemia |
|  | Cardiomyopathies | Structurally and functionally impaired myocardial functioning, excluding ischemic cardiomyopathy and in absence of coronary artery disease, hypertension, valvular disease and congenital heart disease to explain the abnormality (for example dilated cardiomyopathy or hypertrophic cardiomyopathy) |
|  | Valvular abnormality | Insufficiency or stenosis of the aortic, mitral, pulmonic and/or tricuspid valve |
|  | Heart failure | Clinical syndrome with symptoms and/or signs caused by structural and/or functional cardiac abnormality |
|  | Cardiopulmonary diseases | Pulmonary disorders that negatively impact cardiovascular functioning (for example pulmonary embolism and pulmonary hypertension) |
| **Musculoskeletal domain** | Physical activity | Any bodily activity produced by skeletal muscles (exercise, recreational or occupational activities) |
|  | Muscle strength | The maximal force of skeletal muscles on exertion |
|  | Gait/Balance performance | The ability to walk and the ability to maintain balance with minimal postural sway. |
|  | Fear of falling | A person's anxiety towards usual or normal walking or mobilizing, with the perception that a fall will occur |
| **Extrinsic factors** | Alcohol | Alcohol ingestion |
|  | Temperature | Body and/or environment temperature |
|  | Sedatives/hypnotics | Including anxiolytics (ATC class N05B) and hypnotics and sedatives (ATC class N05C). |
|  | Antidepressants | Including selective serotonin reuptake inhibitors (SSRIs), tricyclic antidepressants (TCAs), monoamine oxidase inhibitors (MAOIs), serotonin and norepinephrine reuptake inhibitors (SNRIs), tetracyclic antidepressants and other antidepressants; ATC classes N06A and N06AX |
|  | Antipsychotics | (A)typical antipsychotics; ATC class N05A |
|  | Cholinesterase inhibitors | For example, donepezil and rivastigmine; ATC class N06DA |
|  | Parkinson medication | Drugs used in the treatment of Parkinson disease and related conditions, for example levodopa (ATC class N04) |
|  | Class I antiarrhythmics | Preparations used in the treatment of arrhythmias. Class I agents interfere with the sodium (Na+) channels- membrane-stabilizing agents, for example flecainide; ATC class C01B |
|  | Class III antiarrhythmics | Preparations used in the treatment of arrhythmias. Class III agents interfere with the potassium channels, for example amiodaron; ATC class C01B |
|  | Cardiac glycosides | For example: digoxin (ATC class C01A) |
|  | Cardiac vasodilators | Including drugs used in ischemic heart diseases (ATC class C01D), for example nitrates |
|  | Alpha blocking agents | For example, doxazosin; ATC class C02CA |
|  | Beta blocking agents | For example, metoprolol; ATC class C07 |
|  | ACE inhibitors/ARB | For example, captopril and losartan; ATC classes C09AA and C09CA |
|  | Calcium channel blockers | Dihydropyridines (for example amlodipine, nifedipine) and non-dihydropyridines (for example verapamil); ATC classes C08C, C08D and C08E |
|  | Diuretics | For example, furosemide, hydrochlorothiazide and spironolactone; ATC classes C03A to C03X |
|  | Statins | HMG CoA reductase inhibitors, for example simvastatin; ATC class C10AA |

**Supplementary Table 3** The causal loop diagram connections with references to the literature support.

| **Domain** | **Origin** | **Polarity** | **Destination** | **Literature support** |
| --- | --- | --- | --- | --- |
| **Key variables** | Orthostatic Hypotension | dotted (+) | Microvascular cerebral lesions | Aoki [1] (2013); Buckley [2] (2020); Colloby [3] (2011) |
|  | Orthostatic Hypotension | - | Cerebral blood flow | Claassen [4] (2021); Novak [5] (1998) |
|  | Orthostatic Hypotension | - | CO2 level | Stewart [6] (2012) |
|  | Orthostatic Hypotension | - | Baroreflex activity | Kaufman [7] (2020) |
|  | Orthostatic Hypotension | + | Vasopressin | Zerbe [8] (1983); Torabi [9] (2020) |
|  | Orthostatic Hypotension | - | Muscle Strength | Mol [10] (2018) |
|  | Falls | + | Fear of falling | Chen [11] (2021); Miller [12] (2009) |
| **Cerebral domain** | Cerebral autoregulation | + | Cerebral vascular resistance | Claassen [4] (2021); Payne [13](2016); Numan [14] (2014) |
|  | Cerebral vascular resistance | - | Cerebral blood flow | Claassen [4] (2021); Payne [13] (2016) |
|  | Cerebral blood flow | + | Cerebral oxygenation | Johnston [15] (2003) |
|  | Cerebral blood flow | + | Neurocognitive functioning | Claassen [4] (2021) |
|  | Cerebral blood flow | - | CO2 level | Iovino [16] (2013) |
|  | Cerebral blood flow | dotted (-) | Microvascular cerebral lesions | Claassen [4] (2021); van Buchem [17] (2014) |
|  | Cerebral oxygenation | + | Baroreflex activity | McBryde [18] (2017) |
|  | Cerebral oxygenation | - | Falls | Fitzgibbon-Collins [19] (2021); Kharraziha [20] (2019) |
|  | Cerebral oxygenation | + | Neurocognitive functioning | Claassen [4] (2021); Caine [21] (2000); Leeuwis [22] (2018) |
|  | Cerebral oxygenation | dotted (-) | Microvascular cerebral lesions | Kaufman [23] (2009); Wang [24] (2018) |
|  | Cerebral oxygenation | + | Gait /balance performance | O'Connor [25] (2022) |
|  | Autonomic function | - | Baroreflex activity | Kaufman [7] (2020) |
|  | Autonomic function | dotted (+) | Intravascular volume status | Biaggioni [26] (2007) |
|  | Autonomic function | dotted (-) | Cerebral vascular resistance | van Lieshout [27] (2008); Strandgaard [28] (2008); Levine [29] (2008); ter Laan [30] (2013) |
|  | Neurocognitive functioning | + | Gait /balance performance | Waite [31] (2005); Best [32] (2016); Ansai [33] (2017); Amboni [34] (2013) |
|  | Neurocognitive functioning | + | Physical activity | Hartman [35] (2018) |
|  | Neurocognitive functioning | - | Fear of falling | Soysal [36] (2021); MacKay [37] (2021); Kasai [38] (2017) |
|  | Neurocognitive functioning | - | Falls | Zhang [39] (2019) |
|  | Parkinson(-ism) | - | Gait /balance performance | Chen [40] (2020); Kim [41] (2017) |
|  | Parkinson(-ism) | - | Autonomic function | Chen [40] (2020); Kim [41] (2017) |
|  | Parkinson(-ism) | - | Neurocognitive functioning | Watson [42] (2010) |
|  | Parkinson(-ism) | + | Parkinson medication | Armstrong [43] (2020) |
|  | CO2 level | - | Cerebral vascular resistance | Claassen [44] (2007); van Lieshout [45] (2003) |
|  | CO2 level | - | Cerebral autoregulation | Claassen [4] (2021); Ogoh [46] (2019); van Lieshout [45] (2003) |
|  | CO2 level | + | Vasopressin | Iovino [16] (2013) |
|  | Microvascular cerebral lesions | - | Gait /balance performance | Srikanth [47] (2009); Aoki [1] (2013) |
|  | Microvascular cerebral lesions | - | Neurocognitive functioning | van den Berg [48] (2018); Kloppenborg [49] (2014);  Liu [50] (2018); Akoudad [51] (2016); Gorelick [52] (2011) |
|  | Microvascular cerebral lesions | dotted (-) | Cerebral blood flow | Mozumder [53] (2019) |
| **Cardiovascular  domain** | Cardiac output | + | Physical activity | Pina [54] (2003); |
|  | Cardiac output | - | Orthostatic Hypotension | Gorelik [55] (2016) |
|  | Cardiac output | + | Blood pressure | Mayet [56] (2003) |
|  | Stroke volume | + | Cardiac output | Vincent [57] (2008); |
|  | Heart rate | + | Cardiac output | Vincent [57] (2008) |
|  | Heart rate | D (+/-) | Stroke volume | Wessale [58] (1990) |
|  | Blood pressure | - | Stroke volume | Bruss [59] (2021) |
|  | Blood pressure | - | Vasopressin | den Ouden [60] (2005) |
|  | Blood pressure | + | Cerebral autoregulation | Claassen [4] (2021); Payne [13] (2016); Numan [14] (2014) |
|  | Blood pressure | + | Cerebral Blood flow | Claassen [4] (2021); Payne [13] (2016) |
|  | Blood pressure | + | Baroreflex activity | Kaufman [7] (2020) |
|  | Blood pressure | dotted (-) | Microvascular cerebral lesions | Kim [61] (2020) |
|  | Baroreflex activity | + | Vasodilatation | Kaufman [7] (2020) |
|  | Baroreflex activity | + | Orthostatic Hypotension | Ziegler [62] (2018); Freeman [63] (2018) |
|  | Baroreflex activity | - | Heart rate | Kaufman [7] (2020) |
|  | Baroreflex activity | dotted (-) | Cerebral autoregulation | Claassen [4] (2021); Ogoh [46] (2019) |
|  | Venous pooling | - | Stroke volume | Freeman [64] (2011); Tansey [65] (2019) |
|  | Arterial stiffness | - | Cerebral autoregulation | Claassen [4] (2021); van Buchem [17] (2014) |
|  | Arterial stiffness | + | Baroreflex activity | Mattace-Raso [66] (2007); Ziegler [62] (2018) |
|  | Arterial stiffness | + | Orthostatic Hypotension | Boddaert [67] (2004); Mattace-Raso [66] (2007); Takahashi [68] (2015); Ziegler [62] (2018) |
|  | Arterial stiffness | + | Microvascular cerebral lesions | Ohmine [69] (2008); Bots [70] (1993); |
|  | Brady-/tachyarrhythmia | + | Microvascular cerebral lesions | Kalantarian [71] (2014); de Leeuw [72] (2000) |
|  | Brady-/tachyarrhythmia | + | Heart failure | Perez-Silva [73] (2009) |
|  | Brady-/tachyarrhythmia | - | Stroke volume | Hayashi [74] (2019); Klabunde [75] (2022; Hemodynamic Consequences of Arrhythmias) |
|  | Brady-/tachyarrhythmia | + | Cardiomyopathy | Perez-Silva [73] (2009) |
|  | Vasopressin | + | Baroreflex activity | Hasser [76] (1997) |
|  | Vasopressin | + | Intravascular volume status | Ball [77] (2007) |
|  | Intravascular volume status | + | Blood pressure | Klabunde [78] (2014; blood volume) |
|  | Vasodilation | + | Orthostatic Hypotension | Rivasi [79] (2020) |
|  | Vasodilation | - | Blood pressure | Siddiqui [80] (2011) |
|  | Vasodilation | + | Venous pooling | Tansey [65] (2019), Gelman [81] (2008) |
|  | Hypertension | + | Arterial stiffness | Iadecola [82] (2008); Iadecola [83] (2016) |
|  | Hypertension | + | Microvascular cerebral lesions | Iadecola [83] (2016); van Dijk [84] (2004); Walker [85] (2017) |
|  | Hypertension | + | Ischemic heart diseases | Wright [86] (2015); Vasan [87] (2001) |
|  | Hypertension | + | Valvular abnormality | Saeed [88] (2020); Rahimi [89] (2018) |
|  | Hypertension | + | Heart failure | Lloyd-Jones [90] (2002); Slivnick [91] (2019) |
|  | Heart failure | - | Stroke volume | Klabunde [92] (2015; pathofysiology of heart failure); Kemp [93] (2012) |
|  | Heart failure | + | Heart rate | Hori [94] (2012) |
|  | Heart failure | + | Cardiopulmonary diseases | Krishnan [95] (2022) |
|  | Heart failure | + | Brady-/tachy arrhythmia | Masarone [96] (2017) |
|  | Cardiomyopathy | - | Stroke volume | Klabunde [92] (2015; Klabundeof heart failure); Bruss [59] (2021) |
|  | Cardiomyopathy | + | Heart failure | [Seferovic](https://pubmed.ncbi.nlm.nih.gov/?term=Seferovi%C4%87+PM&cauthor_id=30989768) [97] (2019) |
|  | Cardiomyopathy | + | Valvular abnormality | Schwammenthal [98] (1998); Goel [99] (2009) |
|  | Cardiomyopathy | + | Brady-/tachy arrhythmia | Kumar [100] (2015); O'Mahony [101] (2013) |
|  | Valvular abnormality | - | Stroke volume | Klabunde [102] (Valvular stenosis) |
|  | Valvular abnormality | + | Brady-/tachy arrhythmia | Darby [103] (2012) |
|  | Valvular abnormality | + | Heart failure | Kemp [93] (2012) |
|  | Ischemic heart diseases | - | Stroke volume | Klabunde [92] (pathofysiology of heart failure); Kemp [93] (2012) |
|  | Ischemic heart diseases | + | Valvular abnormality | Varma [104] (2017); Matta [105] (2019) |
|  | Ischemic heart diseases | + | Brady-/tachyarrhythmia | Ghuran [106] (2001) |
|  | Ischemic heart diseases | + | Heart failure | Vedin [107] (2017); Remme [108] (2000) |
|  | Cardiopulmonary diseases | + | Brady-/tachyarrhythmia | Wanamaker [109] (2018) |
|  | Cardiopulmonary diseases | + | Heart failure | Han [110] (2007) |
|  | Cardiopulmonary diseases | - | Stroke volume | Han [110] (2007) |
| **Musculoskeletal domain** | Gait /balance performance | + | Physical activity | Halvarsson [111] (2015); Bai [112] (2022) |
|  | Gait /balance performance | - | Falls | Sherrington [113] (2019) |
|  | Physical activity | + | Heart rate | Nystoriak [114] (2018) |
|  | Physical activity | - | Hypertension | Nystoriak [114] (2018); Diaz [115] (2013) |
|  | Physical activity | + | Muscle Strength | Hamer [116] (2013); Hazell [117] (2007) |
|  | Physical activity | + | Gait /balance performance | Bai [112] (2022); de Labra [118] (2015) |
|  | Muscle Strength | - | Venous pooling | Krediet [119] (2002); Xu [120] (2020) |
|  | Muscle Strength | - | Falls | Landi [121] (2012); Moreland [122] (2004) |
|  | Muscle Strength | - | Orthostatic Hypotension | Benton [123] (2020); Soysal [124] (2020) |
|  | Fear of falling | - | Physical activity | Miller [12] (2009); Kendrick [125] (2014) |
| **Extrinsic factors** | Antipsychotics | + | Brady-/tachyarrhythmia | Li [126] (2021); Stroup [127] (2018); |
|  | Antipsychotics | + | Parkinson(-ism) | Stroup [127] (2018); |
|  | Antipsychotics | + | Arterial stiffness | Findikli [128] (2016); Fiedorowicz [129] (2012); Pillinger [130] (2020) |
|  | Antipsychotics | + | Vasodilation | Khasawneh [131] (2014) |
|  | Antipsychotics | + | Heart rate | Stroup [127] (2018) |
|  | Antipsychotics | D (+/-) | Neurocognitive functioning | Stroup [127] (2018); Woodward [132] (2005); Calsolaro [133] (2019) |
|  | Antipsychotics | - | Gait /balance performance | Stroup [127] (2018) |
|  | Antidepressants | D (+/-) | Neurocognitive functioning | Alagiakrishnan [134] (2004); van Poelgeest [135] (2021); Baune [136] (2018); Rosenblat [137] (2015) |
|  | Antidepressants | + | Parkinson(-ism) | Revet [138] (2020) |
|  | Antidepressants | - | Gait/balance performance | Van Poelgeest [135] (2021); Donoghue [139] (2015). |
|  | Antidepressants | + | Vasodilation | Mago [140] (2014); Calvi [141] (2021) |
|  | Antidepressants | + | Heart rate | Kemp [142] (2014) |
|  | Antidepressants | + | Brady-/tachyarrhythmia | Van Poelgeest [135] (2021) |
|  | Sedatives/hypnotics | D (+/-) | Neurocognitive functioning | Holbrook [143] (2000); Chen [144] (2016); Stewart [145] (2005) |
|  | Sedatives/hypnotics | + | Vasodilation | Rivasi [79] (2020) |
|  | Sedatives/hypnotics | - | Gait/balance performance | Allain [146] (2005); Gray [147] (2006) |
|  | Cholinesterase inhibitors | + | Brady/tachyarrhythmias | Turan Isik [148] (2018); Huang [149] (2020) |
|  | Cholinesterase inhibitors | + | Neurocognitive functioning | Rolinski [150] (2012) |
|  | Cholinesterase inhibitors | D (+/-) | Gait/balance performance | Positive: Turan Isik [151] (2016); Chen [152] (2021); Montero-Odasso [153] (2015);  Negative: Imbimbo [154] (2012) |
|  | Cholinesterase inhibitors | + | Hypertension | Turan Isik  [148] (2018) |
|  | Cholinesterase inhibitors | - | Blood pressure | Turan Isik [148] (2018) Press [155] (2021) |
|  | Parkinson medication | - | Parkinson(-ism) | McNeely [156] (2012) |
|  | Parkinson medication | D (+/-) | Neurocognitive functioning | Negative: Wood [157] (2010)- dopamine receptor agonist, Goldenberg [158] (2008)-levodopa.  Positive: Ikeda [159] (2017) |
|  | Parkinson medication | D (+/-) | Gait/balance performance | Negative: Goldenberg [158] (2008); Curtze [160] (2015); Thanvi [161] (2007) Positive: McNeely [156] (2012); Curtze [160] (2015). |
|  | Parkinson medication | + | Brady/tachyarrhythmias | Goldberg [162] (1971); Goldenberg [158] (2008). |
|  | Parkinson medication | + | Vasodilation | Wood [157] (2010); Goldberg [162] (1971); Goldenberg [158] (2008) |
|  | Type I antiarrhythmic | - | Gait/balance performance | Paolini [163] (2019); Kumar [164] (2021); Tamargo [165] (2012) |
|  | Type I antiarrhythmic | D (+/-) | Brady/tachyarrhythmias | Negative: Andrikopoulos [166] (2015); Paolini [163] (2019) Positive: Paolini [163] (2019) |
|  | Type I antiarrhythmic | - | stroke volume | Paolini [163] (2019) |
|  | Type I antiarrhythmic | + | heart failure | Andrikopoulos [166] (2015) |
|  | Type III antiarrhythmic | D (+/-) | Brady/tachyarrhythmias | Negative/positive: Srinivasan [167] (2019) |
|  | Type III antiarrhythmic | - | Gait/balance performance | Srinivasan [167] (2019); Stanton [168] (2020) |
|  | Type III antiarrhythmic | - | Heart rate | Srinivasan [167] (2019) |
|  | Type III antiarrhythmic | - | blood pressure | Doshi [169] (2015); Zanetti [170] (1993); Khan [171] (2003) |
|  | Cardiac glycosides | - | Gait/balance performance | Patocka [172] (2020); Eichhorn [173] (2002) |
|  | Cardiac glycosides | D (+/-) | Brady/tachyarrhythmias | Patocka [172] (2020); Eichhorn [173] (2002) |
|  | Cardiac glycosides | - | Heart rate | Patocka [172] (2020) |
|  | Cardiac glycosides | + | stroke volume | Patocka [172] (2020) |
|  | Cardiac vasodilators | - | Ischemic heart disease | Giuseppe [174] (2015); Abrams [175] (1996); Thadani [176] (2006) |
|  | Cardiac vasodilators | + | Stroke volume | Klabunde [177] (nitrodilators) |
|  | Cardiac vasodilators | + | Vasodilation | Giuseppe [174] (2015), Abrams [175] (1996), Thadani [176] (2006) |
|  | Alpha blocking agents | + | Vasodilation | Klabunde [177] (Alpha-Adrenoceptor Antagonists) |
|  | Alpha blocking agents | - | hypertension | Klabunde [178] (Alpha-Adrenoceptor Antagonists); Wykretowicz [179] (2008); Williams [180] (2018) |
|  | Beta-blocking agents | - | Neurocognitive functioning | Goldner [181] (2012); Chang [182] (2013); Gliebus [183] (2007); |
|  | Beta-blocking agents | - | Gait/balance performance | Ladage [184] (2012) |
|  | Beta-blocking agents | - | Blood pressure | Wong [185] (2016); Williams [180] (2018); Klabunde [186] (Beta-Adrenoceptor Antagonists) |
|  | Beta-blocking agents | - | Brady/tachyarrhythmias | Hindricks [187] (2020); Brugada [188] (2019); Klabunde [186] (Beta-Adrenoceptor Antagonists) |
|  | Beta-blocking agents | - | Heart rate | Klabunde [186] (Beta-Adrenoceptor Antagonists) |
|  | Beta-blocking agents | - | hypertension | Williams [180] (2018) |
|  | Beta-blocking agents | D (+/-) | Heart failure | Ko [189] (2004); McDonagh [190] (2021) |
|  | Calcium channel blockers:  dihydropyridine | dotted (+) | Gait/balance performance | Beaton [191] (2002) |
|  | Calcium channel blockers:  dihydropyridine | + | Brady/tachyarrhythmias | Eroglu [192] (2020); Russel [193] (1988) |
|  | Calcium channel blockers:  dihydropyridine | - | hypertension | Williams [180] (2018) |
|  | Calcium channel blockers:  dihydropyridine | + | Vasodilation | Russel [193] (1988); Klabunde [194] (Calcium-Channel Blockers) |
|  | Calcium channel blockers:  non-dihydropyridine | dotted (+) | Gait/balance performance | Matsumura [195] (2009) |
|  | Calcium channel blockers:  non-dihydropyridine | - | Brady/tachyarrhythmias | Brugada [188] (2019); Madias [196] (uptodate) |
|  | Calcium channel blockers:  non-dihydropyridine | + | Vasodilation | Russel [193](1988); Klabunde [194] (Calcium-Channel Blockers) |
|  | Calcium channel blockers:  non-dihydropyridine | - | stroke volume | Russel [193] (1988); Klabunde [194] (Calcium-Channel Blockers) |
|  | Calcium channel blockers:  non-dihydropyridine | - | heart rate | Klabunde [194] (Calcium-Channel Blockers) |
|  | Diuretics | - | Gait/balance performance | de Groot [197] (2013); Weinberger [198] (1988), Nakano [199] (2020) |
|  | Diuretics | - | Intravascular volume status | Gifford [200] (1961); Duarte [201] (2010); Shah [202] (2004) |
|  | Diuretics | - | Muscle Strentgh | Weinberger [198] (1988), Nakano [199] (2020) |
|  | Diuretics | + | Brady/tachyarrhythmias | Weinberger [198] (1988) |
|  | Diuretics | - | hypertension | Shah [202] (2004) |
|  | Diuretics | + | Vasodilation | Duarte [201] (2010); Dormans [203] (1996); Shah [202] (2004) |
|  | Diuretics | - | Heart failure | McDonagh [190] (2021) |
|  | Statins | - | Gait /balance performance | Golomb [204] (2012); Joy [205] (2009) |
|  | ACE inhibitors/ARBs | + | Vasodilation | Ma [206] (2010) |
|  | ACE inhibitors/ARBs | - | Intravascular volume status | Ma [206] (2010) |
|  | ACE inhibitors/ARBs | - | Heart failure | McDonagh [190] (2021), Sayer [207] (2014) |
|  | ACE inhibitors/ARBs | - | hypertension | Williams [180] (2018) |
|  | ACE inhibitors/ARBs | + | stroke volume | Klabunde [208] (Angiotensin Converting Enzyme (ACE) Inhibitors); Khalil [209] (2001) |
|  | ACE inhibitors/ARBs | D (+/-)  dotted | Gait /balance performance | Sumukadas [210] (2008); Dutta [211] (2001) |
|  | Alcohol | - | Gait /balance performance | Sullivan [212] (1995) |
|  | Alcohol | - | Intravascular volume status | Polhuis [213] (2017) |
|  | Alcohol | - | Neurocognitive functioning | Brust [214] (2010); Xu [215] (2019) |
|  | Alcohol | + | Vasodilation | Narkiewicz [216] (2000) |
|  | Alcohol | + | cardiomyopathy | Maisch [217] (2016) |
|  | Temperature | + | Vasopressin | Iovino [16] (2013) |
|  | Temperature | + | Vasodilatation | Schlader [218] (2016); Charkoudian [219] (2010) |
|  | Temperature | - | Intravascular volume status | Schlader [218] (2016) |

**Supplementary Table 4** Overview of feedback loops in the causal loop diagram.

| **Domain** | **Feedback loops** | **1st variable** | **2nd variable** | **3rd variable** | **4th variable** | **5th variable** |
| --- | --- | --- | --- | --- | --- | --- |
| **Cerebral  domain** | B1 | Cerebral oxygenation (+) | Baroreflex activity (+) | Orthostatic hypotension (-) | Cerebral blood flow (+) |  |
|  | H1 (B) | Cerebral oxygenation (+) | Baroreflex activity (+) | Orthostatic hypotension (dotted +) | Microvascular cerebral lesions (dotted -) | Cerebral blood flow (+) |
|  | B2 | Parkinson(-ism) (+) | Parkinson medication (-) |  |  |  |
|  | R1 | Cerebral blood flow (+) | Neurocognitive functioning (+) | Physical activity (+) | Muscle strength (-) | Orthostatic hypotension (-) |
|  | H2 (R) | Cerebral blood flow (dotted -) | Microvascular cerebral lesions (dotted -) |  |  |  |
|  | H3 (R) | Cerebral blood flow (+) | Neurocognitive functioning (+) | Physical activity  (-) | Hypertension (+) | Microvascular cerebral lesions (dotted -) |
|  | B3 | Cerebral blood flow (+) | Cerebral oxygenation (+) | Baroreflex activity (+) | Vasodilation (+) | Orthostatic hypotension (-) |
|  | H4 (R) | Cerebral blood flow (+) | Cerebral oxygenation (dotted -) | Microvascular cerebral lesions (dotted -) |  |  |
|  | B4 | Cerebral blood flow (-) | CO2 level (-) | Cerebral vascular resistance (-) |  |  |
|  | B5 | Cerebral blood flow (-) | CO2 level (-) | Cerebral autoregulation (+) | Cerebral vascular resistance (-) |  |
|  | H5 (R) | Microvascular cerebral lesions (-) | Neurocognitive functioning (+) | Physical activity (+) | Muscle strength (-) | Orthostatic hypotension (dotted +) |
|  | R2 | Microvascular cerebral lesions (-) | Neurocognitive functioning (+) | Physical activity (-) | Hypertension (+) |  |
|  | R3 | Microvascular cerebral lesions (-) | Neurocognitive functioning (+) | Physical activity (-) | Hypertension (+) | Arterial stiffness (+) |
|  | R4 | Microvascular cerebral lesions (-) | Neurocognitive functioning (+) | Gait/balance performance (+) | Physical activity (-) | Hypertension (+) |
|  | R5 | Microvascular cerebral lesions (-) | Neurocognitive functioning (-) | Fear of falling  (-) | Physical activity (-) | Hypertension (+) |
|  | H6 (R) | Cerebral autoregulation (+) | Cerebral vascular resistance (-) | Cerebral blood flow (+) | Cerebral oxygenation (+) | Baroreflex activity (dotted -) |
|  | B6 | CO2 level (+) | Vasopressin (+) | Baroreflex activity (+) | Orthostatic hypotension  (-) |  |
|  | R6 | CO2 level (+) | Vasopressin (+) | Baroreflex activity (+) | Orthostatic hypotension  (-) | Cerebral blood flow (-) |
|  | B7 | CO2 level (+) | Vasopressin (+) | Baroreflex activity (+) | Vasodilation (+) | Orthostatic hypotension (-) |
|  | B8 | CO2 level (+) | Vasopressin (+) | Intravascular volume status (+) | Blood pressure (+) | Cerebral blood flow (-) |
| **Cardiovascular  domain** | B9 | Baroreflex activity (+) | Orthostatic hypotension (-) |  |  |  |
|  | B10 | Baroreflex activity (+) | Vasodilation (+) | Orthostatic hypotension (-) |  |  |
|  | R7 | Baroreflex activity (+) | Vasodilation (+) | Orthostatic hypotension (+) | Vasopressin (+) |  |
|  | B11 | Baroreflex activity (+) | Vasodilation (-) | Blood pressure (+) | Cerebral blood flow (+) | Cerebral oxygenation (+) |
|  | R8 | Baroreflex activity (+) | Orthostatic hypotension (+) | Vasopressin (+) | Intravascular volume status (+) | Blood pressure (+) |
|  | R9 | Vasopressin (+) | Baroreflex activity (+) | Orthostatic hypotension (+) |  |  |
|  | B12 | Vasopressin (+) | Intravascular volume status (+) | Blood pressure (-) |  |  |
|  | B13 | Vasopressin (+) | Baroreflex activity (+) | Heart rate (+) | Cardiac output (-) | Orthostatic hypotension (+) |
|  | R10 | Vasopressin (+) | Baroreflex activity (+) | Vasodilation (-) | Blood pressure (-) |  |
|  | B14 | Blood pressure (+) | Baroreflex activity (+) | Vasodilation (-) |  |  |
|  | B15 | Blood pressure (+) | Baroreflex activity (-) | Heart rate (+) | Cardiac output (+) |  |
|  | B16 | Blood pressure (-) | Stroke volume (+) | Cardiac output (+) |  |  |
|  | R11 | Heart rate (+) | Cardiac output (+) | Physical activity (+) |  |  |
|  | B17 | Heart rate (+) | Cardiac output (-) | Orthostatic hypotension (-) | Baroreflex activity (-) |  |
|  | R12 | Heart rate (+) | Cardiac output (-) | Orthostatic hypotension (+) | Vasopressin (+) | Baroreflex activity (-) |
|  | R13 | Heart rate (+) | Cardiac output (+) | Blood pressure (-) | Vasopressin (+) | Baroreflex activity (-) |
|  | R14 | Heart rate (-) | Stroke volume (+) | Cardiac output (-) | Orthostatic hypotension  (-) | Baroreflex activity (-) |
|  | B18 | Heart rate (+) | Stroke volume (+) | Cardiac output (-) | Orthostatic hypotension  (-) | Baroreflex activity (-) |
|  | B19 | Heart rate (-) | Stroke volume (+) | Cardiac output (+) | Physical activity (-) |  |
|  | R15 | Heart rate (+) | Stroke volume (+) | Cardiac output (+) | Physical activity (+) |  |
|  | R16 | Heart rate (-) | Stroke volume (+) | Cardiac output (+) | Blood pressure (+) | Baroreflex activity (-) |
|  | B20 | Heart rate (+) | Stroke volume (+) | Cardiac output (+) | Blood pressure (+) | Baroreflex activity (-) |
|  | B21 | Heart rate (+) | Cardiac output (+) | Physical activity  (-) | Hypertension (+) | Heart failure (+) |
|  | R17 | Stroke volume (+) | Cardiac output (+) | Physical activity  (-) | Hypertension (+) | Heart failure (-) |
|  | R18 | Cardiopulmonary diseases (+) | Heart failure (+) |  |  |  |
|  | R19 | Brady-tachyarrhythmia (+) | Cardiomyopathy (+) |  |  |  |
|  | R20 | Brady-tachyarrhythmia (+) | Heart failure (+) |  |  |  |
|  | R21 | Brady-tachyarrhythmia (+) | Heart failure (+) | Cardiopulmonary diseases (+) |  |  |
|  | R22 | Brady-tachyarrhythmia (+) | Cardiomyopathy (+) | Heart failure (+) |  |  |
|  | R23 | Brady-tachyarrhythmia (+) | Cardiomyopathy (+) | Heart failure (+) | Cardiopulmonary diseases (+) |  |
|  | R24 | Brady-tachyarrhythmia (+) | Cardiomyopathy (+) | Valvular abnormality (+) |  |  |
|  | R25 | Brady-tachyarrhythmia (+) | Cardiomyopathy (+) | Valvular abnormality (+) | Heart failure (+) |  |
|  | R26 | Brady-tachyarrhythmia (+) | Cardiomyopathy (+) | Valvular abnormality (+) | Heart failure (+) | Cardiopulmonary diseases (+) |
|  | R27 | Hypertension (+) | Valvular abnormality (-) | Stroke volume (+) | Cardiac output (+) | Physical activity (-) |
|  | R28 | Hypertension (+) | Ischemic heart diseases (-) | Stroke volume (+) | Cardiac output (+) | Physical activity (-) |
| **Musculoskeletal domain** | R29 | Muscle strength (-) | Orthostatic hypotension (-) |  |  |  |
|  | R30 | Muscle strength (-) | Falls (+) | Fear of falling  (-) | Physical activity (+) |  |
|  | R31 | Muscle strength (-) | Venous pooling  (-) | Stroke volume (+) | Cardiac output **(-)** | Orthostatic hypotension (-) |
|  | R32 | Physical activity (+) | Gait/balance performance (+) |  |  |  |
|  | R33 | Physical activity (+) | Heart rate (+) | Cardiac output (+) |  |  |
|  | R34 | Physical activity (+) | Muscle strength (-) | Venous pooling (-) | Stroke volume (+) | Cardiac output (+) |
|  | R35 | Gait/balance performance (+) | Physical activity (-) | Hypertension (+) | Microvascular cerebral lesions (-) |  |
|  | R36 | Gait/balance performance (+) | Physical activity (-) | Hypertension (+) | Arterial stiffness (+) | Microvascular cerebral lesions (-) |
|  | H7 (R) | Gait/balance performance (+) | Physical activity (+) | Muscle strength (-) | Orthostatic hypotension (dotted +) | Microvascular cerebral lesions (-) |
|  | R37 | Fear of falling (-) | Physical activity (+) | Gait performance/balance performance (-) | Falls (+) |  |

* Note: key variables (in blue), variables from cerebral domains (in red), cardiovascular domains (in green), musculoskeletal domains (in orange), extrinsic variables (in grey). Reinforcing feedback loops are indicated with the ‘R’, balancing feedback loops with the ‘B’, and feedback loops contained hypothesized (dotted) connections with the ‘H’. The numbers refer to specifically identified loops. Regarding polarity, e.g., Loop R29, Muscle strength (-), Orthostatic hypotension (-), means the polarity from muscle strength 🡪 orthostatic hypotension is negative (-); from orthostatic hypotension🡪 muscle strength is negative (-).

**Reference list for all CLD connections (Supplementary Table 3)**

[1] Aoki M, Tanaka K, Wakaoka T et al. *The association between impaired perception of verticality and cerebral white matter lesions in the elderly patients with orthostatic hypotension*. J Vestib Res 2013;**23**:85-93.

[2] Buckley A, Carey D, Meaney JM, Kenny R, Harbison J. *Is there an association between orthostatic hypotension and cerebral white matter hyperintensities in older people? The Irish longitudinal study on ageing*. JRSM Cardiovasc Dis 2020;**9**:2048004020954628.

[3] Colloby SJ, Vasudev A, O'Brien JT et al. *Relationship of orthostatic blood pressure to white matter hyperintensities and subcortical volumes in late-life depression*. Br J Psychiatry 2011;**199**:404-10.

[4] Claassen J, Thijssen DHJ, Panerai RB, Faraci FM. *Regulation of cerebral blood flow in humans: physiology and clinical implications of autoregulation*. Physiol Rev 2021;**101**:1487-559.

[5] Novak V, Novak P, Spies JM, Low PA. *Autoregulation of cerebral blood flow in orthostatic hypotension*. Stroke 1998;**29**:104-11.

[6] Stewart JM. *Mechanisms of sympathetic regulation in orthostatic intolerance*. J Appl Physiol (1985) 2012;**113**:1659-68.

[7] Kaufmann H, Norcliffe-Kaufmann L, Palma JA. *Baroreflex Dysfunction*. N Engl J Med 2020;**382**:163-78.

[8] Zerbe RL, Henry DP, Robertson GL. *Vasopressin response to orthostatic hypotension. Etiologic and clinical implications*. Am J Med 1983;**74**:265-71.

[9] Torabi P, Ricci F, Hamrefors V, Sutton R, Fedorowski A. *Classical and Delayed Orthostatic Hypotension in Patients With Unexplained Syncope and Severe Orthostatic Intolerance*. Front Cardiovasc Med 2020;**7**:21.

[10] Mol A, Reijnierse EM, Bui Hoang PTS et al. *Orthostatic hypotension and physical functioning in older adults: A systematic review and meta-analysis*. Ageing Res Rev 2018;**48**:122-44.

[11] Chen WC, Li YT, Tung TH, Chen C, Tsai CY. *The relationship between falling and fear of falling among community-dwelling elderly*. Medicine (Baltimore) 2021;**100**:e26492.

[12] Miller RR, Ballew SH, Shardell MD et al. *Repeat falls and the recovery of social participation in the year post-hip fracture*. Age Ageing 2009;**38**:570-5.

[13] Payne S. *Cerebral autoregulation: control of blood flow in the brain*. 2016.

[14] Numan T, Bain AR, Hoiland RL et al. *Static autoregulation in humans: a review and reanalysis*. Med Eng Phys 2014;**36**:1487-95.

[15] Johnston AJ, Steiner LA, Gupta AK, Menon DK. *Cerebral oxygen vasoreactivity and cerebral tissue oxygen reactivity*. Br J Anaesth 2003;**90**:774-86.

[16] Iovino M, Guastamacchia E, Giagulli VA et al. *Role of central and peripheral chemoreceptors in vasopressin secretion control*. Endocr Metab Immune Disord Drug Targets 2013;**13**:250-5.

[17] van Buchem MA, Biessels GJ, Brunner la Rocca HP et al. *The heart-brain connection: a multidisciplinary approach targeting a missing link in the pathophysiology of vascular cognitive impairment*. J Alzheimers Dis 2014;**42 Suppl 4**:S443-51.

[18] McBryde FD, Malpas SC, Paton JF. *Intracranial mechanisms for preserving brain blood flow in health and disease*. Acta Physiol (Oxf) 2017;**219**:274-87.

[19] Fitzgibbon-Collins LK, Heckman GA, Bains I et al. *Older Adults' Drop in Cerebral Oxygenation on Standing Correlates With Postural Instability and May Improve With Sitting Prior to Standing*. J Gerontol A Biol Sci Med Sci 2021;**76**:1124-33.

[20] Kharraziha I, Holm H, Bachus E et al. *Cerebral Oximetry in Syncope and Syndromes of Orthostatic Intolerance*. Front Cardiovasc Med 2019;**6**:171.

[21] Caine D, Watson JD. *Neuropsychological and neuropathological sequelae of cerebral anoxia: a critical review*. J Int Neuropsychol Soc 2000;**6**:86-99.

[22] Leeuwis AE, Smith LA, Melbourne A et al. *Cerebral Blood Flow and Cognitive Functioning in a Community-Based, Multi-Ethnic Cohort: The SABRE Study*. Front Aging Neurosci 2018;**10**:279.

[23] Kaufman JL, Karceski S. *Risk factors and prevention of lacunar infarcts in 60- to 64-year-olds*. Neurology 2009;**73**:e17-9.

[24] Wang F, Cao Y, Ma L et al. *Dysfunction of Cerebrovascular Endothelial Cells: Prelude to Vascular Dementia*. Front Aging Neurosci 2018;**10**:376.

[25] O'Connor JD, O'Connell MDL, Knight SP et al. *Impaired Stabilization of Orthostatic Cerebral Oxygenation Is Associated With Slower Gait Speed: Evidence From The Irish Longitudinal Study on Ageing*. J Gerontol A Biol Sci Med Sci 2022;**77**:1216-21.

[26] Biaggioni I. *The sympathetic nervous system and blood volume regulation: lessons from autonomic failure patients*. Am J Med Sci 2007;**334**:61-4.

[27] van Lieshout JJ, Secher NH. *Point:Counterpoint: Sympathetic activity does/does not influence cerebral blood flow. Point: Sympathetic activity does influence cerebral blood flow*. J Appl Physiol (1985) 2008;**105**:1364-6.

[28] Strandgaard S, Sigurdsson ST. *Point:Counterpoint: Sympathetic activity does/does not influence cerebral blood flow. Counterpoint: Sympathetic nerve activity does not influence cerebral blood flow*. J Appl Physiol (1985) 2008;**105**:1366-7; discussion 67-8.

[29] Levine BD, Zhang R. *Comments on Point:Counterpoint: Sympathetic activity does/does not influence cerebral blood flow*. Journal of Applied Physiology 2008;**105**:1369-73.

[30] ter Laan M, van Dijk JM, Elting JW, Staal MJ, Absalom AR. *Sympathetic regulation of cerebral blood flow in humans: a review*. Br J Anaesth 2013;**111**:361-7.

[31] Waite LM, Grayson DA, Piguet O et al. *Gait slowing as a predictor of incident dementia: 6-year longitudinal data from the Sydney Older Persons Study*. J Neurol Sci 2005;**229-230**:89-93.

[32] Best JR, Liu-Ambrose T, Boudreau RM et al. *An Evaluation of the Longitudinal, Bidirectional Associations Between Gait Speed and Cognition in Older Women and Men*. J Gerontol A Biol Sci Med Sci 2016;**71**:1616-23.

[33] Ansai JH, Andrade LP, Nakagawa TH et al. *Cognitive Correlates of Timed Up and Go Subtasks in Older People With Preserved Cognition, Mild Cognitive Impairment, and Alzheimer's Disease*. Am J Phys Med Rehabil 2017;**96**:700-05.

[34] Amboni M, Barone P, Hausdorff JM. *Cognitive contributions to gait and falls: evidence and implications*. Mov Disord 2013;**28**:1520-33.

[35] Hartman YAW, Karssemeijer EGA, van Diepen LAM, Olde Rikkert MGM, Thijssen DHJ. *Dementia Patients Are More Sedentary and Less Physically Active than Age- and Sex-Matched Cognitively Healthy Older Adults*. Dement Geriatr Cogn Disord 2018;**46**:81-89.

[36] Soysal P, Tan SG, Smith L. *A comparison of the prevalence of Fear of Falling between older patients with Lewy body dementia, Alzheimer's disease, and without dementia*. Exp Gerontol 2021;**146**:111248.

[37] MacKay S, Ebert P, Harbidge C, Hogan DB. *Fear of Falling in Older Adults: A Scoping Review of Recent Literature*. Can Geriatr J 2021;**24**:379-94.

[38] Kasai M, Meguro K, Ozawa H et al. *Fear of Falling and Cognitive Impairments in Elderly People with Hip Fractures*. Dement Geriatr Cogn Dis Extra 2017;**7**:386-94.

[39] Zhang W, Low LF, Schwenk M et al. *Review of Gait, Cognition, and Fall Risks with Implications for Fall Prevention in Older Adults with Dementia*. Dement Geriatr Cogn Disord 2019;**48**:17-29.

[40] Chen Z, Li G, Liu J. *Autonomic dysfunction in Parkinson's disease: Implications for pathophysiology, diagnosis, and treatment*. Neurobiol Dis 2020;**134**:104700.

[41] Kim JS, Ryu DW, Oh JH et al. *Cardiovascular Autonomic Dysfunction in Patients with Drug-Induced Parkinsonism*. J Clin Neurol 2017;**13**:15-20.

[42] Watson GS, Leverenz JB. *Profile of cognitive impairment in Parkinson's disease*. Brain Pathol 2010;**20**:640-5.

[43] Armstrong MJ, Okun MS. *Diagnosis and Treatment of Parkinson Disease: A Review*. JAMA 2020;**323**:548-60.

[44] Claassen JA, Zhang R, Fu Q, Witkowski S, Levine BD. *Transcranial Doppler estimation of cerebral blood flow and cerebrovascular conductance during modified rebreathing*. J Appl Physiol (1985) 2007;**102**:870-7.

[45] Van Lieshout JJ, Wieling W, Karemaker JM, Secher NH. *Syncope, cerebral perfusion, and oxygenation*. J Appl Physiol (1985) 2003;**94**:833-48.

[46] Ogoh S, Tarumi T. *Cerebral blood flow regulation and cognitive function: a role of arterial baroreflex function*. J Physiol Sci 2019;**69**:813-23.

[47] Srikanth V, Beare R, Blizzard L et al. *Cerebral white matter lesions, gait, and the risk of incident falls: a prospective population-based study*. Stroke 2009;**40**:175-80.

[48] van den Berg E, Geerlings MI, Biessels GJ, Nederkoorn PJ, Kloppenborg RP. *White Matter Hyperintensities and Cognition in Mild Cognitive Impairment and Alzheimer's Disease: A Domain-Specific Meta-Analysis*. J Alzheimers Dis 2018;**63**:515-27.

[49] Kloppenborg RP, Nederkoorn PJ, Geerlings MI, van den Berg E. *Presence and progression of white matter hyperintensities and cognition: a meta-analysis*. Neurology 2014;**82**:2127-38.

[50] Liu Y, Braidy N, Poljak A, Chan DKY, Sachdev P. *Cerebral small vessel disease and the risk of Alzheimer's disease: A systematic review*. Ageing Res Rev 2018;**47**:41-48.

[51] Akoudad S, Wolters FJ, Viswanathan A et al. *Association of Cerebral Microbleeds With Cognitive Decline and Dementia*. JAMA Neurol 2016;**73**:934-43.

[52] Gorelick PB, Scuteri A, Black SE et al. *Vascular contributions to cognitive impairment and dementia: a statement for healthcare professionals from the american heart association/american stroke association*. Stroke 2011;**42**:2672-713.

[53] Mozumder M, Pozo JM, Coelho S et al. *Quantitative histomorphometry of capillary microstructure in deep white matter*. Neuroimage Clin 2019;**23**:101839.

[54] Pina IL, Apstein CS, Balady GJ et al. *Exercise and heart failure: A statement from the American Heart Association Committee on exercise, rehabilitation, and prevention*. Circulation 2003;**107**:1210-25.

[55] Gorelik O, Feldman L, Cohen N. *Heart failure and orthostatic hypotension*. Heart Fail Rev 2016;**21**:529-38.

[56] Mayet J, Hughes A. *Cardiac and vascular pathophysiology in hypertension*. Heart 2003;**89**:1104-9.

[57] Vincent JL. *Understanding cardiac output*. Crit Care 2008;**12**:174.

[58] Wessale JL, Voelz MB, Geddes LA. *Stroke volume and the three phase cardiac output rate relationship with ventricular pacing*. Pacing Clin Electrophysiol 1990;**13**:673-80.

[59] Bruss. ZS, Raja. A. *Physiology, Stroke Volum*. 2022.

[60] den Ouden DT, Meinders AE. *Vasopressin: physiology and clinical use in patients with vasodilatory shock: a review*. Neth J Med 2005;**63**:4-13.

[61] Kim JS, Lee S, Suh SW et al. *Association of Low Blood Pressure with White Matter Hyperintensities in Elderly Individuals with Controlled Hypertension*. J Stroke 2020;**22**:99-107.

[62] Ziegler MG. *Atherosclerosis and Blood Pressure Variability*. Hypertension 2018;**71**:403-05.

[63] Freeman R, Abuzinadah AR, Gibbons C et al. *Orthostatic Hypotension: JACC State-of-the-Art Review*. J Am Coll Cardiol 2018;**72**:1294-309.

[64] Freeman R, Wieling W, Axelrod FB et al. *Consensus statement on the definition of orthostatic hypotension, neurally mediated syncope and the postural tachycardia syndrome*. Clin Auton Res 2011;**21**:69-72.

[65] Tansey EA, Montgomery LEA, Quinn JG, Roe SM, Johnson CD. *Understanding basic vein physiology and venous blood pressure through simple physical assessments*. Adv Physiol Educ 2019;**43**:423-29.

[66] Mattace-Raso FU, van den Meiracker AH, Bos WJ et al. *Arterial stiffness, cardiovagal baroreflex sensitivity and postural blood pressure changes in older adults: the Rotterdam Study*. J Hypertens 2007;**25**:1421-6.

[67] Boddaert J, Tamim H, Verny M, Belmin J. *Arterial stiffness is associated with orthostatic hypotension in elderly subjects with history of falls*. J Am Geriatr Soc 2004;**52**:568-72.

[68] Takahashi M, Miyai N, Nagano S et al. *Orthostatic Blood Pressure Changes and Subclinical Markers of Atherosclerosis*. Am J Hypertens 2015;**28**:1134-40.

[69] Ohmine T, Miwa Y, Yao H et al. *Association between arterial stiffness and cerebral white matter lesions in community-dwelling elderly subjects*. Hypertens Res 2008;**31**:75-81.

[70] Bots ML, van Swieten JC, Breteler MM et al. *Cerebral white matter lesions and atherosclerosis in the Rotterdam Study*. Lancet 1993;**341**:1232-7.

[71] Kalantarian S, Ay H, Gollub RL et al. *Association between atrial fibrillation and silent cerebral infarctions: a systematic review and meta-analysis*. Ann Intern Med 2014;**161**:650-8.

[72] de Leeuw FE, de Groot JC, Oudkerk M et al. *Atrial fibrillation and the risk of cerebral white matter lesions*. Neurology 2000;**54**:1795-801.

[73] A. Pérez-Silva, Merino. J. Tachycardia induced Cardiomyopathy. *E-Journal*, 2009.

[74] Hayashi H, Abe Y, Morita Y et al. *Impact of stroke volume on prognostic outcome in patients with atrial fibrillation and concomitant heart failure with preserved ejection fraction*. J Cardiol 2019;**73**:307-12.

[75] Klabunde RE. *Cardiovascular Physiology Concepts. Topic: Hemodynamic Consequences of Arrhythmias.* <https://www.cvphysiology.com/Arrhythmias/A011> (1 May, 2022 date last accessed).

[76] Hasser EM, Bishop VS, Hay M. *Interactions between vasopressin and baroreflex control of the sympathetic nervous system*. Clin Exp Pharmacol Physiol 1997;**24**:102-8.

[77] Ball SG. *Vasopressin and disorders of water balance: the physiology and pathophysiology of vasopressin*. Ann Clin Biochem 2007;**44**:417-31.

[78] Klabunde RE. *Cardiovascular Physiology Concepts. Topic: Blood volume.* <https://www.cvphysiology.com/Blood%20Pressure/BP025> (1 May, 2022 date last accessed).

[79] Rivasi G, Rafanelli M, Mossello E, Brignole M, Ungar A. *Drug-Related Orthostatic Hypotension: Beyond Anti-Hypertensive Medications*. Drugs Aging 2020;**37**:725-38.

[80] Siddiqui A. *Effects of Vasodilation and Arterial Resistance on Cardiac Output*. Journal of Clinical & Experimental Cardiology 2011;**02**.

[81] Gelman S. *Venous function and central venous pressure: a physiologic story*. Anesthesiology 2008;**108**:735-48.

[82] Iadecola C, Davisson RL. *Hypertension and cerebrovascular dysfunction*. Cell Metab 2008;**7**:476-84.

[83] Iadecola C, Yaffe K, Biller J et al. *Impact of Hypertension on Cognitive Function: A Scientific Statement From the American Heart Association*. Hypertension 2016;**68**:e67-e94.

[84] van Dijk EJ, Breteler MM, Schmidt R et al. *The association between blood pressure, hypertension, and cerebral white matter lesions: cardiovascular determinants of dementia study*. Hypertension 2004;**44**:625-30.

[85] Walker KA, Power MC, Gottesman RF. *Defining the Relationship Between Hypertension, Cognitive Decline, and Dementia: a Review*. Curr Hypertens Rep 2017;**19**:24.

[86] Wright JT, Jr., Group SR, Williamson JD et al. *A Randomized Trial of Intensive versus Standard Blood-Pressure Control*. N Engl J Med 2015;**373**:2103-16.

[87] Vasan RS, Larson MG, Leip EP et al. *Impact of high-normal blood pressure on the risk of cardiovascular disease*. N Engl J Med 2001;**345**:1291-7.

[88] Saeed S, Scalise F, Chambers JB, Mancia G. *Hypertension in aortic stenosis: a focused review and recommendations for clinical practice*. J Hypertens 2020;**38**:1211-19.

[89] Rahimi K, Mohseni H, Kiran A et al. *Elevated blood pressure and risk of aortic valve disease: a cohort analysis of 5.4 million UK adults*. Eur Heart J 2018;**39**:3596-603.

[90] Lloyd-Jones DM, Larson MG, Leip EP et al. *Lifetime risk for developing congestive heart failure: the Framingham Heart Study*. Circulation 2002;**106**:3068-72.

[91] Slivnick J, Lampert BC. *Hypertension and Heart Failure*. Heart Fail Clin 2019;**15**:531-41.

[92] Klabunde RE. *Cardiovascular Physiology Concepts. Topic: Pathophysiology of Heart Failure.* <https://www.cvphysiology.com/Heart%20Failure/HF003> (1 May, 2022 date last accessed).

[93] Kemp CD, Conte JV. *The pathophysiology of heart failure*. Cardiovasc Pathol 2012;**21**:365-71.

[94] Hori M, Okamoto H. *Heart rate as a target of treatment of chronic heart failure*. J Cardiol 2012;**60**:86-90.

[95] Krishnan U, Horn E. *Pulmonary hypertension due to left heart disease (group 2 pulmonary hypertension) in adults*. Uptodate 2022.

[96] Masarone D, Limongelli G, Rubino M et al. *Management of Arrhythmias in Heart Failure*. J Cardiovasc Dev Dis 2017;**4**.

[97] Seferovic PM, Polovina M, Bauersachs J et al. *Heart failure in cardiomyopathies: a position paper from the Heart Failure Association of the European Society of Cardiology*. Eur J Heart Fail 2019;**21**:553-76.

[98] Schwammenthal E, Nakatani S, He S et al. *Mechanism of mitral regurgitation in hypertrophic cardiomyopathy: mismatch of posterior to anterior leaflet length and mobility*. Circulation 1998;**98**:856-65.

[99] Goel R, Sengupta PP, Mookadam F et al. *Valvular regurgitation and stenosis: when is surgery required?* Heart Asia 2009;**1**:20-5.

[100] Kumar S, Stevenson WG, John RM. *Arrhythmias in dilated cardiomyopathy*. Card Electrophysiol Clin 2015;**7**:221-33.

[101] O'Mahony C, Elliott P, McKenna W. *Sudden cardiac death in hypertrophic cardiomyopathy*. Circ Arrhythm Electrophysiol 2013;**6**:443-51.

[102] Klabunde RE. *Cardiovascular Physiology Concepts. Topic: Valvular stenosis.* <https://www.cvphysiology.com/Heart%20Disease/HD004> (1 May, 2022 date last accessed).

[103] Darby AE, Dimarco JP. *Management of atrial fibrillation in patients with structural heart disease*. Circulation 2012;**125**:945-57.

[104] Varma PK, Krishna N, Jose RL, Madkaiker AN. *Ischemic mitral regurgitation*. Ann Card Anaesth 2017;**20**:432-39.

[105] Matta A, Moussallem N. *Coronary artery disease is associated with valvular heart disease, but could it Be a predictive factor?* Indian Heart J 2019;**71**:284-87.

[106] Ghuran AV, Camm AJ. *Ischaemic heart disease presenting as arrhythmias*. Br Med Bull 2001;**59**:193-210.

[107] Vedin O, Lam CSP, Koh AS et al. *Significance of Ischemic Heart Disease in Patients With Heart Failure and Preserved, Midrange, and Reduced Ejection Fraction: A Nationwide Cohort Study*. Circ Heart Fail 2017;**10**.

[108] Remme WJ. *Overview of the relationship between ischemia and congestive heart failure*. Clin Cardiol 2000;**23**:IV4-8.

[109] Wanamaker B, Cascino T, McLaughlin V et al. *Atrial Arrhythmias in Pulmonary Hypertension: Pathogenesis, Prognosis and Management*. Arrhythm Electrophysiol Rev 2018;**7**:43-48.

[110] Han MK, McLaughlin VV, Criner GJ, Martinez FJ. *Pulmonary diseases and the heart*. Circulation 2007;**116**:2992-3005.

[111] Halvarsson A, Franzen E, Stahle A. *Balance training with multi-task exercises improves fall-related self-efficacy, gait, balance performance and physical function in older adults with osteoporosis: a randomized controlled trial*. Clin Rehabil 2015;**29**:365-75.

[112] Bai X, Soh KG, Omar Dev RD et al. *Aerobic Exercise Combination Intervention to Improve Physical Performance Among the Elderly: A Systematic Review*. Front Physiol 2021;**12**:798068.

[113] Sherrington C, Fairhall NJ, Wallbank GK et al. *Exercise for preventing falls in older people living in the community*. Cochrane Database Syst Rev 2019;**1**:CD012424.

[114] Nystoriak MA, Bhatnagar A. *Cardiovascular Effects and Benefits of Exercise*. Front Cardiovasc Med 2018;**5**:135.

[115] Diaz KM, Shimbo D. *Physical activity and the prevention of hypertension*. Curr Hypertens Rep 2013;**15**:659-68.

[116] Hamer M, Stamatakis E. *Screen-based sedentary behavior, physical activity, and muscle strength in the English longitudinal study of ageing*. PLoS One 2013;**8**:e66222.

[117] Hazell T, Kenno K, Jakobi J. *Functional benefit of power training for older adults*. J Aging Phys Act 2007;**15**:349-59.

[118] de Labra C, Guimaraes-Pinheiro C, Maseda A, Lorenzo T, Millan-Calenti JC. *Effects of physical exercise interventions in frail older adults: a systematic review of randomized controlled trials*. BMC Geriatr 2015;**15**:154.

[119] Krediet CT, van Dijk N, Linzer M, van Lieshout JJ, Wieling W. *Management of vasovagal syncope: controlling or aborting faints by leg crossing and muscle tensing*. Circulation 2002;**106**:1684-9.

[120] Xu D, Tremblay MF, Verma AK et al. *Cardio-postural interactions and muscle-pump baroreflex are severely impacted by 60-day bedrest immobilization*. Sci Rep 2020;**10**:12042.

[121] Landi F, Liperoti R, Russo A et al. *Sarcopenia as a risk factor for falls in elderly individuals: results from the ilSIRENTE study*. Clin Nutr 2012;**31**:652-8.

[122] Moreland JD, Richardson JA, Goldsmith CH, Clase CM. *Muscle weakness and falls in older adults: a systematic review and meta-analysis*. J Am Geriatr Soc 2004;**52**:1121-9.

[123] Benton MJ, Silva-Smith AL, Spicher JM. *Muscle Loss is Associated with Risk of Orthostatic Hypotension in Older Men and Women*. J Frailty Aging 2021;**10**:219-25.

[124] Soysal P, Kocyigit SE, Dokuzlar O et al. *Relationship between sarcopenia and orthostatic hypotension*. Age Ageing 2020;**49**:959-65.

[125] Kendrick D, Kumar A, Carpenter H et al. *Exercise for reducing fear of falling in older people living in the community*. Cochrane Database Syst Rev 2014;**2014**:CD009848.

[126] Li XQ, Tang XR, Li LL. *Antipsychotics cardiotoxicity: What's known and what's next*. World J Psychiatry 2021;**11**:736-53.

[127] Stroup TS, Gray N. *Management of common adverse effects of antipsychotic medications*. World Psychiatry 2018;**17**:341-56.

[128] Findikli E, Gokce M, Nacitarhan V et al. *Arterial Stiffness in Patients Taking Second-generation Antipsychotics*. Clin Psychopharmacol Neurosci 2016;**14**:365-70.

[129] Fiedorowicz JG, Coryell WH, Rice JP, Warren LL, Haynes WG. *Vasculopathy related to manic/hypomanic symptom burden and first-generation antipsychotics in a sub-sample from the collaborative depression study*. Psychother Psychosom 2012;**81**:235-43.

[130] Pillinger T, McCutcheon RA, Vano L et al. *Comparative effects of 18 antipsychotics on metabolic function in patients with schizophrenia, predictors of metabolic dysregulation, and association with psychopathology: a systematic review and network meta-analysis*. Lancet Psychiatry 2020;**7**:64-77.

[131] Khasawneh FT, Shankar GS. *Minimizing cardiovascular adverse effects of atypical antipsychotic drugs in patients with schizophrenia*. Cardiol Res Pract 2014;**2014**:273060.

[132] Woodward ND, Purdon SE, Meltzer HY, Zald DH. *A meta-analysis of neuropsychological change to clozapine, olanzapine, quetiapine, and risperidone in schizophrenia*. Int J Neuropsychopharmacol 2005;**8**:457-72.

[133] Calsolaro V, Antognoli R, Okoye C, Monzani F. *The Use of Antipsychotic Drugs for Treating Behavioral Symptoms in Alzheimer's Disease*. Front Pharmacol 2019;**10**:1465.

[134] Alagiakrishnan K, Wiens CA. *An approach to drug induced delirium in the elderly*. Postgrad Med J 2004;**80**:388-93.

[135] van Poelgeest EP, Pronk AC, Rhebergen D, van der Velde N. *Depression, antidepressants and fall risk: therapeutic dilemmas-a clinical review*. Eur Geriatr Med 2021;**12**:585-96.

[136] Baune BT, Brignone M, Larsen KG. *A Network Meta-Analysis Comparing Effects of Various Antidepressant Classes on the Digit Symbol Substitution Test (DSST) as a Measure of Cognitive Dysfunction in Patients with Major Depressive Disorder*. Int J Neuropsychopharmacol 2018;**21**:97-107.

[137] Rosenblat JD, Kakar R, McIntyre RS. *The Cognitive Effects of Antidepressants in Major Depressive Disorder: A Systematic Review and Meta-Analysis of Randomized Clinical Trials*. Int J Neuropsychopharmacol 2015;**19**.

[138] Revet A, Montastruc F, Roussin A et al. *Antidepressants and movement disorders: a postmarketing study in the world pharmacovigilance database*. BMC Psychiatry 2020;**20**:308.

[139] Donoghue OA, O'Hare C, King-Kallimanis B, Kenny RA. *Antidepressants are independently associated with gait deficits in single and dual task conditions*. Am J Geriatr Psychiatry 2015;**23**:189-99.

[140] Mago R, Tripathi N, Andrade C. *Cardiovascular adverse effects of newer antidepressants*. Expert Rev Neurother 2014;**14**:539-51.

[141] Calvi A, Fischetti I, Verzicco I et al. *Antidepressant Drugs Effects on Blood Pressure*. Front Cardiovasc Med 2021;**8**:704281.

[142] Kemp AH, Brunoni AR, Santos IS et al. *Effects of depression, anxiety, comorbidity, and antidepressants on resting-state heart rate and its variability: an ELSA-Brasil cohort baseline study*. Am J Psychiatry 2014;**171**:1328-34.

[143] Holbrook AM, Crowther R, Lotter A, Cheng C, D. K. *Meta-analysis of benzodiazepine use in the treatment of insomnia*. CMAJ, 2000.

[144] Chen L, Bell JS, Visvanathan R et al. *The association between benzodiazepine use and sleep quality in residential aged care facilities: a cross-sectional study*. BMC Geriatr 2016;**16**:196.

[145] Stewart SA. *The effects of benzodiazepines on cognition*. J Clin Psychiatry 2005;**66 Suppl 2**:9-13.

[146] Allain H, Bentue-Ferrer D, Polard E, Akwa Y, Patat A. *Postural instability and consequent falls and hip fractures associated with use of hypnotics in the elderly: a comparative review*. Drugs Aging 2005;**22**:749-65.

[147] Gray SL, LaCroix AZ, Hanlon JT et al. *Benzodiazepine use and physical disability in community-dwelling older adults*. J Am Geriatr Soc 2006;**54**:224-30.

[148] Isik AT, Soysal P, Stubbs B et al. *Cardiovascular Outcomes of Cholinesterase Inhibitors in Individuals with Dementia: A Meta-Analysis and Systematic Review*. J Am Geriatr Soc 2018;**66**:1805-11.

[149] Huang Y, Alsabbagh MW. *Comparative risk of cardiac arrhythmias associated with acetylcholinesterase inhibitors used in treatment of dementias - A narrative review*. Pharmacol Res Perspect 2020;**8**:e00622.

[150] Rolinski M, Fox C, Maidment I, McShane R. *Cholinesterase inhibitors for dementia with Lewy bodies, Parkinson's disease dementia and cognitive impairment in Parkinson's disease*. Cochrane Database Syst Rev 2012;**2012**:CD006504.

[151] Isik AT, Soysal P, Usarel C. *Effects of Acetylcholinesterase Inhibitors on Balance and Gait Functions and Orthostatic Hypotension in Elderly Patients With Alzheimer Disease*. Am J Alzheimers Dis Other Demen 2016;**31**:580-84.

[152] Chen JH, Huang TW, Hong CT. *Cholinesterase inhibitors for gait, balance, and fall in Parkinson disease: a meta-analysis*. NPJ Parkinsons Dis 2021;**7**:103.

[153] Montero-Odasso M, Muir-Hunter SW, Oteng-Amoako A et al. *Donepezil improves gait performance in older adults with mild Alzheimer's disease: a phase II clinical trial*. J Alzheimers Dis 2015;**43**:193-9.

[154] Imbimbo BP. *Pharmacodynamic-tolerability relationships of cholinesterase inhibitors for Alzheimer's disease*. CNS Drugs 2001;**15**:375-90.

[155] Press. D, Alexander M. *Cholinesterase inhibitors in the treatment of dementia.* <https://www.uptodate.com/contents/cholinesterase-inhibitors-in-the-treatment-of-dementia#H2076322556> (1 May, 2022 date last accessed).

[156] McNeely ME, Duncan RP, Earhart GM. *Medication improves balance and complex gait performance in Parkinson disease*. Gait Posture 2012;**36**:144-8.

[157] Wood LD. *Clinical review and treatment of select adverse effects of dopamine receptor agonists in Parkinson's disease*. Drugs Aging 2010;**27**:295-310.

[158] Goldenberg MM. *Medical Management of Parkinson’s Disease*. P&T 2008;**33**:590-606.

[159] Ikeda M KH, Ueno S. *Can levodopa prevent cognitive decline in patients with Parkinson’s disease?* . American Journal of Neurodegenerative Disease 2017;**6**:9–14.

[160] Curtze C, Nutt JG, Carlson-Kuhta P, Mancini M, Horak FB. *Levodopa Is a Double-Edged Sword for Balance and Gait in People With Parkinson's Disease*. Mov Disord 2015;**30**:1361-70.

[161] Thanvi B, Lo N, Robinson T. *Levodopa-induced dyskinesia in Parkinson's disease: clinical features, pathogenesis, prevention and treatment*. Postgrad Med J 2007;**83**:384-8.

[162] Goldberg LI WT. *Cardiovascular effects of levodopa*. JAMA 1971;**218**:1921-3.

[163] Paolini E, Stronati G, Guerra F, Capucci A. *Flecainide: Electrophysiological properties, clinical indications, and practical aspects*. Pharmacol Res 2019;**148**:104443.

[164] Kumar D, Rehman ME, Mersfelder T, Patel P. *Flecainide-induced myalgias and weakness: a rare adverse reaction*. BMJ Case Rep 2021;**14**.

[165] Tamargo J, Capucci A, Mabo P. *Safety of flecainide*. Drug Saf 2012;**35**:273-89.

[166] Andrikopoulos GK, Pastromas S, Tzeis S. *Flecainide: Current status and perspectives in arrhythmia management*. World J Cardiol 2015;**7**:76-85.

[167] Srinivasan M, Ahmad L, Bhindi R, Allahwala U. *Amiodarone in the aged*. Aust Prescr 2019;**42**:158-62.

[168] Stanton MM, Samii L, Leung G, Pearce P. *Amiodarone-induced neuromyopathy in a geriatric patient*. BMJ Case Rep 2020;**13**.

[169] Doshi D, Jayawardana R. *Amiodarone-Induced Life-Threatening Refractory Hypotension*. Am J Case Rep 2015;**16**:617-20.

[170] Zanetti LA. *Sotalol: a new class III antiarrhythmic agent*. Clin Pharm 1993;**12**:883-91.

[171] Khan IA, Mehta NJ, Gowda RM. *Amiodarone for pharmacological cardioversion of recent-onset atrial fibrillation*. Int J Cardiol 2003;**89**:239-48.

[172] Patocka J, Nepovimova E, Wu W, Kuca K. *Digoxin: Pharmacology and toxicology-A review*. Environ Toxicol Pharmacol 2020;**79**:103400.

[173] Eichhorn EJ, Gheorghiade M. *Digoxin. Progress in Cardiovascular Diseases*. Prog Cardiovasc Dis 2002;**44**:251-66.

[174] Giuseppe C, Paul J, Hans-Ulrich I. *Use of nitrates in ischemic heart disease*. Expert Opin Pharmacother 2015;**16**:1567-72.

[175] Abrams J. *Beneficial actions of nitrates in cardiovascular disease*. Am J Cardiol 1996;**77**:31C-7C.

[176] Thadani U, Rodgers T. *Side effects of using nitrates to treat angina*. Expert Opin Drug Saf 2006;**5**:667-74.

[177] Klabunde RE. *Cardiovascular pharmacology concepts. Topic: Nitrodilators.* <https://cvpharmacology.com/vasodilator/nitro> (1 May, 2022 date last accessed).

[178] Klabunde RE. *Cardiovascular pharmacology concepts. Topic: Alpha-Adrenoceptor Antagonists (Alpha-Blockers).* <https://cvpharmacology.com/vasodilator/alpha> (1 May, 2022 date last accessed).

[179] Wykretowicz A, Guzik P, Wysocki H. *Doxazosin in the current treatment of hypertension*. Expert Opin Pharmacother 2008;**9**:625-33.

[180] Williams B, Mancia G, Spiering W et al. *2018 ESC/ESH Guidelines for the management of arterial hypertension*. Eur Heart J 2018;**39**:3021-104.

[181] Goldner JA. *Metoprolol-induced visual hallucinations: a case series*. J Med Case Rep 2012;**6**:65.

[182] Chang CH, Yang YH, Lin SJ et al. *Risk of insomnia attributable to beta-blockers in elderly patients with newly diagnosed hypertension*. Drug Metab Pharmacokinet 2013;**28**:53-8.

[183] Gliebus G, Lippa CF. *The influence of beta-blockers on delayed memory function in people with cognitive impairment*. Am J Alzheimers Dis Other Demen 2007;**22**:57-61.

[184] Ladage D, Schwinger RH, Brixius K. *Cardio-selective beta-blocker: pharmacological evidence and their influence on exercise capacity*. Cardiovasc Ther 2013;**31**:76-83.

[185] Wong GW, Boyda HN, Wright JM. *Blood pressure lowering efficacy of beta-1 selective beta blockers for primary hypertension*. Cochrane Database Syst Rev 2016;**3**:CD007451.

[186] Klabunde RE. *Cardiovascular pharmacology concepts. Topic: Beta-Adrenoceptor Antagonists (Beta-Blockers).* <https://www.cvpharmacology.com/cardioinhibitory/beta-blockers> (1 May, 2022 date last accessed).

[187] Hindricks G, Potpara T, Dagres N et al. *2020 ESC Guidelines for the diagnosis and management of atrial fibrillation developed in collaboration with the European Association for Cardio-Thoracic Surgery (EACTS): The Task Force for the diagnosis and management of atrial fibrillation of the European Society of Cardiology (ESC) Developed with the special contribution of the European Heart Rhythm Association (EHRA) of the ESC*. Eur Heart J 2021;**42**:373-498.

[188] Brugada J, Katritsis DG, Arbelo E et al. *2019 ESC Guidelines for the management of patients with supraventricular tachycardiaThe Task Force for the management of patients with supraventricular tachycardia of the European Society of Cardiology (ESC)*. Eur Heart J 2020;**41**:655-720.

[189] Ko DT, Hebert PR, Coffey CS et al. *Adverse effects of beta-blocker therapy for patients with heart failure: a quantitative overview of randomized trials*. Arch Intern Med 2004;**164**:1389-94.

[190] McDonagh TA, Metra M, Adamo M et al. *2021 ESC Guidelines for the diagnosis and treatment of acute and chronic heart failure*. Eur Heart J 2021;**42**:3599-726.

[191] Beaton LJ, Tarnopolsky MA, Phillips SM. *Contraction-induced muscle damage in humans following calcium channel blocker administration*. J Physiol 2002;**544**:849-59.

[192] Eroglu TE, Mohr GH, Blom MT et al. *Differential effects on out-of-hospital cardiac arrest of dihydropyridines: real-world data from population-based cohorts across two European countries*. Eur Heart J Cardiovasc Pharmacother 2020;**6**:347-55.

[193] Russell RP. *Side effects of calcium channel blockers*. Hypertension 1988;**11**:II42-4.

[194] Klabunde RE. *Cardiovascular pharmacology concepts. Topic: Calcium-Channel Blockers (CCBs).* <https://www.cvpharmacology.com/vasodilator/CCB> (1 May, 2022 date last accessed).

[195] Matsumura CY, Pertille A, Albuquerque TC, Santo Neto H, Marques MJ. *Diltiazem and verapamil protect dystrophin-deficient muscle fibers of MDX mice from degeneration: a potential role in calcium buffering and sarcolemmal stability*. Muscle Nerve 2009;**39**:167-76.

[196] Madias C. *Calcium channel blockers in the treatment of cardiac arrhythmias*. Uptodate 2021.

[197] de Groot MH, van Campen JP, Moek MA et al. *The effects of fall-risk-increasing drugs on postural control: a literature review*. Drugs Aging 2013;**30**:901-20.

[198] Weinberger MH. *Diuretics and their side effects. Dilemma in the treatment of hypertension*. Hypertension 1988;**11**:II16-20.

[199] Nakano I, Tsuda M, Kinugawa S et al. *Loop diuretic use is associated with skeletal muscle wasting in patients with heart failure*. J Cardiol 2020;**76**:109-14.

[200] Gifford RW, Jr., Mattox VR, Orvis AL, Sones DA, Rosevear JW. *Effect of thiazide diuretics on plasma volume, body electrolytes, and excretion of aldosterone in hypertension*. Circulation 1961;**24**:1197-205.

[201] Duarte JD, Cooper-DeHoff RM. *Mechanisms for blood pressure lowering and metabolic effects of thiazide and thiazide-like diuretics*. Expert Rev Cardiovasc Ther 2010;**8**:793-802.

[202] Shah SU, Anjum S, Littler WA. *Use of diuretics in cardiovascular disease: (2) hypertension*. Postgrad Med J 2004;**80**:271-6.

[203] Dormans TP, Pickkers P, Russel FG, Smits P. *Vascular effects of loop diuretics*. Cardiovasc Res 1996;**32**:988-97.

[204] Golomb BA, Evans MA, Dimsdale JE, White HL. *Effects of statins on energy and fatigue with exertion: results from a randomized controlled trial*. Arch Intern Med 2012;**172**:1180-2.

[205] Joy TR, Hegele RA. *Narrative review: statin-related myopathy*. Ann Intern Med 2009;**150**:858-68.

[206] Ma TK, Kam KK, Yan BP, Lam YY. *Renin-angiotensin-aldosterone system blockade for cardiovascular diseases: current status*. Br J Pharmacol 2010;**160**:1273-92.

[207] Sayer G, Bhat G. *The renin-angiotensin-aldosterone system and heart failure*. Cardiol Clin 2014;**32**:21-32, vii.

[208] Klabunde RE. *Cardiovascular pharmacology concepts. Topic: Angiotensin Converting Enzyme (ACE) Inhibitors.* <https://www.cvpharmacology.com/vasodilator/ACE> (1 May, 2022 date last accessed).

[209] Khalil ME, Basher AW, Brown EJ, Jr., Alhaddad IA. *A remarkable medical story: benefits of angiotensin-converting enzyme inhibitors in cardiac patients*. J Am Coll Cardiol 2001;**37**:1757-64.

[210] Sumukadas D, Witham MD, Struthers AD, McMurdo ME. *Ace inhibitors as a therapy for sarcopenia - evidence and possible mechanisms*. J Nutr Health Aging 2008;**12**:480-5.

[211] Dutta D, Fischler M, McClung A. *Angiotensin converting enzyme inhibitor induced hyperkalaemic paralysis*. Postgrad Med J 2001;**77**:114-5.

[212] Sullivan EV, Rosenbloom MJ, Deshmukh A, Desmond JE, Pfefferbaum A. *Alcohol and the Cerebellum: Effects on Balance, Motor Coordination, and Cognition*. Alcohol Health Res World 1995;**19**:138-41.

[213] Polhuis K, Wijnen AHC, Sierksma A, Calame W, Tieland M. *The Diuretic Action of Weak and Strong Alcoholic Beverages in Elderly Men: A Randomized Diet-Controlled Crossover Trial*. Nutrients 2017;**9**.

[214] Brust JC. *Ethanol and cognition: indirect effects, neurotoxicity and neuroprotection: a review*. Int J Environ Res Public Health 2010;**7**:1540-57.

[215] Xu K, Montalvo-Ortiz JL, Zhang X et al. *Epigenome-Wide DNA Methylation Association Analysis Identified Novel Loci in Peripheral Cells for Alcohol Consumption Among European American Male Veterans*. Alcohol Clin Exp Res 2019;**43**:2111-21.

[216] Narkiewicz K, Cooley RL, Somers VK. *Alcohol potentiates orthostatic hypotension : implications for alcohol-related syncope*. Circulation 2000;**101**:398-402.

[217] Maisch B. *Alcoholic cardiomyopathy : The result of dosage and individual predisposition*. Herz 2016;**41**:484-93.

[218] Schlader ZJ, Wilson TE, Crandall CG. *Mechanisms of orthostatic intolerance during heat stress*. Auton Neurosci 2016;**196**:37-46.

[219] Charkoudian N. *Mechanisms and modifiers of reflex induced cutaneous vasodilation and vasoconstriction in humans*. J Appl Physiol (1985) 2010;**109**:1221-8.
